# Supplementary material for: Patient perspectives on the HIV continuum of care in London: a qualitative study of people diagnosed between 1986 and 2014
Source: BMJ Open. 2018 Mar 30;8(3):e020208. doi: 10.1136/bmjopen-2017-020208 (PMC5884332; doi:10.1136/bmjopen-2017-020208)
Supplement: Author's manuscript [file bmjopen-2017-020208.draft_revisions.pdf]

BMJ Open is committed to open peer review. As part of this commitment we make the peer review history of every article we publish publicly available.

When an article is published we post the peer reviewers' comments and the authors' responses online. We also post the versions of the paper that were used during peer review. These are the versions that the peer review comments apply to.

The versions of the paper that follow are the versions that were submitted during the peer review process. They are not the versions of record or the final published versions. They should not be cited or distributed as the published version of this manuscript.

BMJ Open is an open access journal and the full, final, typeset and author-corrected version of record of the manuscript is available on our site with no access controls, subscription charges or pay-per-view fees (http://bmjopen.bmj.com).

If you have any questions on BMJ Open's open peer review process please email info.bmjopen@bmj.com

BMJ Open

Patient perspectives on the HIV continuum of care in London: a qualitative study of people diagnosed between 1986 and 2014

Journal:	*BMJ Open*
Manuscript ID	bmjopen-2017-020208
Article Type:	Research
Date Submitted by the Author:	31-Oct-2017
Complete List of Authors:	Bruton, Jane; Imperial College Lodon, Departmnet of Infectious Disease Epidemiology<br>Rai, Tanvi; Imperial College Lodon, Department of Infectious Disease Epidemiology<br>Day, Sophie; Imperial College Lodon, Departmnet of Infectious Disease Epidemiology; Goldsmiths University of London, Anthropology<br>Ward, Helen; Imperial College London, Infectious Disease Epidemiology
**Primary Subject Heading**:	HIV/AIDS
Secondary Subject Heading:	Qualitative research, Health services research, Patient-centred medicine
Keywords:	HIV & AIDS < INFECTIOUS DISEASES, QUALITATIVE RESEARCH, PATIENT EXPERIENCE, HIV CARE CONTINUUM

only

SCHOLARONE™
Manuscripts

For peer review only - http://bmjopen.bmj.com/site/about/guidelines.xhtml

Patient perspectives on the HIV continuum of care in London: a qualitative study of people diagnosed between 1986 and 2014

Jane Bruton RN, BA, MA Medical Anthropology; Tanvi Rai PhD Public Health, MPH, BA, BSc: Sophie Day, PhD, BA, MA Anthropology;, BSc Helen Ward PhD, FRCP, FFPH, MBChB, MSc Epidemiology.

Corresponding author:
Pamela Jane Bruton,
Department of Infectious Disease Epidemiology,
School of Public Health,
Imperial College,
St Marys Campus,
Norfolk Place,
London.
W2 1PG
jbruton@ic.ac.uk
0207 5943464

Co-authors:
Rai, Tanvi: Imperial College London, Department of Infectious Disease Epidemiology.
Day, Sophie; Imperial College London, Department of Infectious Disease Epidemiology and Goldsmiths College
Ward, Helen; Imperial College London, Department of Infectious Disease Epidemiology.

Word count: Main text 3410

For peer review only - http://bmjopen.bmj.com/site/about/guidelines.xhtml

Abstract

Objectives: The care continuum provides a step-wise measure of success in managing HIV. Whilst useful for providers it does not inform us of the actual experience of individuals negotiating the different stages of the HIV care pathway. With the transformation from an acute life-threatening to a chronic long-term condition we hypothesised that the experience of being diagnosed, becoming a patient and starting treatment would have changed over the decades of the epidemic.

Methods: Qualitative interview study of 52 individuals attending two large HIV London clinics, purposively sampled, on the basis of being diagnosed at different stages in the history of the epidemic, into HIV "generations" and analysed thematically.

Results: Some important differences were identified; for earlier generations, the visible illness and deaths from AIDS made it harder to engage with care following diagnosis, likewise subsequent decisions about starting treatment were deeply influenced by the fear of severe side-effects associated with early antiretroviral therapy (ARV). However, despite improvements in ARV and life expectancy over the epidemic we found a striking similarity across the participants' accounts of the key stages of the care continuum, regardless of when they were diagnosed. Diagnosis was a major traumatic life event for almost everyone, fear of testing positive or having low self-perceived risk affected the timing of testing and diagnosis, engaging with care was facilitated by a responsive and flexible approach from services/clinicians and initiating treatment was a major life decision.

Conclusion: A personal and holistic approach has been the hallmark of HIV care since the beginning of the epidemic. It is important that the major advances in biomedical treatment do not lead to an undermining of the care continuum through a loss of care that meets the needs of patients for whom HIV diagnosis and treatment remain significant challenges requiring supportive and flexible care.

For peer review only - http://bmjopen.bmj.com/site/about/guidelines.xhtml

For peer review only

Strengths

- The large number of participants, at two different clinics and the inclusion of people with diverse characteristics and 4 HIV ‘generations’.
- The interview format allowed us to explore factors important to participants rather than asking about pre-defined concerns.

Limitations

- An imbalance between generations, with larger numbers of women in the earliest two generations and only one woman in the recently diagnosed. This means that some of our conclusions about generations may also reflect gendered differences.
- Our recruitment methods meant we were not able to explore the experiences of those who remain outside care.
- Limitation of the single interview format means people were having to recall past experiences, some of which were three decades ago. This may have introduced recall bias with subsequent experience colouring earlier reports.

For peer review only

For peer review only - http://bmjopen.bmj.com/site/about/guidelines.xhtml

INTRODUCTION

The HIV continuum of care provides stepwise estimates for the stages of engagement in care for people living with HIV (PLHIV).[1] The UNAIDS goal to end the AIDS epidemic by 2020 stipulates a target of 90% of all people with HIV diagnosed, of whom 90% are on antiretroviral therapy (ARV), of whom 90% (73% of PLHIV) are virally suppressed.[2] The UK has one of the best outcomes for HIV treatment and care in the world with an estimated 78% of PLHIV having an undetectable viral load.[3, 4] However the statistics do not and cannot tell us about the patient experience of passing through these stages of care whether it be good or bad. The continuum essentially measures the success of programmes from a provider rather than a patient perspective.[5]

Flowers argues that there is a tension between the certainty and confidence of a linear HIV pathway, associated with ideas of clinical efficacy, and patient experiences of diagnosis and prognosis, which can be full of uncertainty.[4] With the evolution of modern ARVs we have witnessed the transformation of HIV from an acute life-threatening to a treatable chronic condition and a concurrent evolving of the care continuum. Analysis of the patients' experiences of passing through each of the stages of care may help to illuminate key factors in the care continuum. This reflection is important in a climate of NHS restructuring in the UK, which has led to reductions in non-clinical services and streamlining of care. To this end we explored the patient perspectives on the care continuum, and hypothesized that patients' experiences of, and engagement with, care would differ according to what point in the epidemic they were diagnosed.

METHODS

We undertook a qualitative study of people attending two large London HIV clinics. We used a purposive sampling method to recruit patients with a range of experiences. To reflect the evolution of antiretroviral therapy (ARV), we identified four 'generations' according to time of diagnosis: pre-1996 (pre-ARV), 1997 to 2005 (complex ARVs), 2006 to 2012 (simpler ARVs), 2013 onwards (recent diagnoses). Within each generation, we aimed to include people with a range of characteristics, including, gender, exposure, age and ethnicity. Participants were recruited opportunistically by researchers attending clinical services, and through fliers and digital advertising in clinical areas. Recruitment was periodically checked against the recruitment matrix and under-represented groups/strata targeted.

Patients were provided with information and gave written consent. Interviews took place in private rooms in or near the clinics or at the patient's home; they were recorded and transcribed, and lasted between 60 and 90 minutes. The interviews were semi-structured and carried out by four researchers (JB, TR, CH, JR) with a topic guide (appended) informed by a focus group of PLHIV who

For peer review only - http://bmjopen.bmj.com/site/about/guidelines.xhtml

assisted in designing the research. We invited participants to recall their initial diagnosis and describe key points in their HIV journey including testing, disclosure, support, engaging with care, starting treatment, medication adherence, work and social life. Field notes were written after the interviews and focus group.

Transcripts were uploaded to NVIVO and analysed using an iterative process of reading and discussing the transcripts, identifying themes and coding the dataset. Final themes were discussed in the research group (HW, JB, SD, TR) and further analysed in relation to the existing literature.

Ethical approval was obtained from NRES (reference number 14/WM/0147) in May 2014, and research governance approval obtained from the local sites.

RESULTS

Fifty-two patients were recruited, 25 at one clinic and 27 at the other. The sample included 41 men and 11 women; 37 men acquired HIV through sex with other men (MSM), the rest through heterosexual contact (14) or injection drug use (1). There were 11 in generation 1, 14 generation 2, 17 generation 3 and 10 generation 4. The characteristics of the study participants alongside those of the clinic population for 2014 are shown in Table 1.

Table 1. Study sample characteristics compared with the cohorts

	Clinic A				Clinic B			
	Study sample		2014 Cohort		Study sample		2014 Cohort	
**Gender**								
Male	18	72.0%	2459	77.5%	23	85.2%	7743	90.3%
Female	7	28.0%	715	22.5%	4	14.8%	830	9.7%
**Age**								
18-24	0	0.0%	124	3.9%	0	0.0%	186	2.2%
25-34	4	16.0%	465	14.7%	3	11.1%	1729	20.2%
35-49	16	64.0%	1502	47.3%	14	51.9%	4164	48.6%
50+	5	20.0%	1083	34.1%	10	37.0%	2494	29.1%
**Ethnicity**								
White	14	56.0%	1541	48.6%	22	81.5%	6401	74.7%
Black African	5	20.0%	720	22.7%	5	18.5%	778	9.1%
Black Caribbean	0	0.0%	114	3.6%	0	0.0%	201	2.3%
Other/Mixed	6	24.0%	746	23.5%	0	0.0%	1132	13.2%
Not reported	0	0.0%	53	1.7%	0	0.0%	61	0.7%
**Exposure route**								
Sex between men	15	60.0%	1971	62.1%	22	81.5%	6776	79.0%
Heterosexual contact	9	36.0%	1037	32.7%	5	18.5%	1209	14.1%

For peer review only - http://bmjopen.bmj.com/site/about/guidelines.xhtml

Injecting drug use	1	4.0%	52	1.6%	0	0.0%	109	1.3%
Other	0	0.0%	112	3.5%	0	0.0%	46	0.5%
Undetermined	0	0.0%	2	0.1%	0	0.0%	433	5.1%
**Year of diagnosis**								
Pre-1997	6	24.0%	638	20.1%	5	18.5%	1399	16.3%
1997-2005	6	24.0%	1234	38.9%	8	29.6%	2580	30.1%
2006-2012	7	28.0%	986	31.1%	10	37.0%	3199	37.3%
2013 onwards	6	24.0%	316	10.0%	4	14.8%	1395	16.3%
Total			3174				8573	

The generation samples differed somewhat by gender and acquisition: the women were concentrated in generations 1 and 2 (6 and 4 respectively), and MSM in generations 3 and 4 (16 and 8).

"Becoming positive" – the impact of HIV diagnosis

The experience of receiving an HIV diagnosis was similar across the generations. First reactions were generally of shock and fear of death, irrespective of generation. Alan (pseudonym), diagnosed in 1991, knew nothing about HIV and had not tested before. He remembers vividly the time he received his result:

> *"I could hear myself saying 'I'm going to die'. Not verbally but in my mind, 'I'm going to die, I'm going to die'"* (A6, Gen1, MSM)

Roger, diagnosed more than 20 years later, reported several previous tests and considered himself well-informed. However, his principal concern on receiving a positive diagnosis was also about life expectancy:

> *"But even I was not certain. Certainty is the wrong word. I was under the illusion that my expiry date was stamped on me now"* (A25, Gen4, MSM)

Fear of a positive result was a factor in delayed diagnosis for several MSM in all generations, who reported concerns about the impact of HIV on their lives. They were aware of their risk, and described feeling relieved at diagnosis as HIV had been *"hanging over them"* for years; the diagnosis confirmed their suspicions. William, who had never tested before, presented with symptoms:

> *"[I had been] burying my head in the sand. I guess I knew I had it but didn't, at the same time, want it confirmed"* (B16, Gen2, MSM)

For peer review only - http://bmjopen.bmj.com/site/about/guidelines.xhtml

For peer review only

Brian (B2, Gen4, MSM), recently diagnosed, had "*spent on and off probably 8 years thinking about it*" He felt he had "*done all the thinking before*" … so although disappointed, he was also relieved.

The response and level of support offered by clinicians at this critical time were important to participants' immediate wellbeing and influenced what happened next, including continuing engagement in care:

> *"I remember how lovely [name of clinician] was and I've always said I could never wish for a better person to ever tell me or try to guide me, or to reassure me more than what she did because she was perfect"* (A24, Gen4, female)

While most experiences were positive, there were some exceptions. Paul (B9, gen3, MSM) had regularly tested negative but continued to take risks. Testing positive in 2010 was totally unexpected, leaving him "*numb with shock*", and he did not feel that he was supported appropriately. The clinician who gave him the diagnosis seemed "[to be on] *autopilot because he had seen people like me before*" and was "*working to his own agenda*". Despite Paul's obvious distress, the clinician asked him to ring potential contacts during the consultation. Further, when the clinician said: "*Oh this can be managed, don't worry*", Paul interpreted this to mean 'managed to his death'. After two weeks of acute anxiety, Paul contacted a friend who was able to reassure him about treatment and the care pathway. Similar experiences led other participants to feel vulnerable, isolated and slow to accept their diagnosis.

"Becoming an HIV patient": Developing a relationship with clinic and clinician

Once diagnosed, participants described a sense of reassurance about being in the "best hands", managed by experts in HIV medicine. The majority across the generations described strong relationships with their clinicians and valued seeing the same person each visit. It felt "*like a partnership*" with "*someone you can tell anything*", who knew them and their entire history, ensuring that care went beyond just the clinical management of HIV: "*We seriously talk about how I am not just what my CD4 count is*".

However, some had not developed a trusting relationship. Marty (B13, gen3, MSM), for example, diagnosed HIV in 2012, was not eligible for treatment under guidelines at that point. He described anxiety about this lack of treatment, feeling that it adversely affected pre-existing mental health problems which were not addressed by his clinicians. He attended two different clinics and was on the verge of dropping out of care when, as he recounted: *"I basically rescued myself*". His friend recommended a clinician: "*She got me just like that thank god, thank god…I finally found and she was willing to fight my case*".

For peer review only - http://bmjopen.bmj.com/site/about/guidelines.xhtml

Another participant, Peter, diagnosed 2009, reported changing HIV clinics within three months of diagnosis. He recalled a series of mistakes, miscommunication and a "dehumanising" clinic environment. Losing trust in clinicians and the service, he finally gained confidence from attending a support group and moved his care

> *"I remember that I said, it was like falling off a building… I'm slowly falling backwards as the virus increases. It felt like they were holding a blanket at the bottom to catch me but it felt like they were holding it in the wrong place. I was being asked to trust"* (B24, Gen3, MSM)

All participants valued continuity of care although two recently diagnosed felt that it was not always necessary to see the same clinician. But, continuity was affected by what some described as the very busy clinics limiting the time for consultations and impeding communication.

All participants were in care at the time of the interview but some described having stayed away in the past. Two of six women diagnosed before 1996 had dropped out of care for several years. Given the lack of effective treatment, they had found clinic visits depressing and preferred to keep away until they became sick. Marie (A8, gen1, female) explained, *"I didn't want a life where I just would go to tests and I am scared and they had nothing to offer".* Alison (B6, gen1, female) described the *"terrible situation"* at the clinic, where she saw young gay men, couples, where one would be fit and the other *"in a wheelchair, a skeleton".* She felt sorry for the doctors - *"there were all these poor young doctors with nothing to offer and seeing these very ill people."*

Seven of all those diagnosed since ARVs became available described occasional or multiple lapses in attendance; these were generally explained by issues external to the clinic such as recreational drug use, household disruption, mental health problems or competing co-morbidities. Re-engagement with care was easier when their clinician actively reached out; for example, some consultants had telephoned patients when they missed appointments and one woman (gen1) described interventions of this kind as being "*like my family*". Even though not all patients were contacted when they did not attend services, and one expressed surprise that no-one had tried, all found their way back into care.

"Becoming medicalised": Starting treatment

Almost all (48) of the participants were on treatment; one had stopped medication because of drug interactions and three chose to remain off treatment. Despite the simplification of regimens, participants in all four generations found the decision to start treatment a significant life event.

For earlier generations, the decision had been complex because of toxicity related to ARVs. Some refused treatment contrary to medical advice. Alison, described earlier, recalled, "*In the waiting*

For peer review only - http://bmjopen.bmj.com/site/about/guidelines.xhtml

rooms people said, 'don't take it, it will kill you', so I refused that (AZT)". Others felt well without treatment like Marie who was diagnosed in 1986 but only started ARVs 25 years after diagnosis when her CD4 count crashed. She felt she had no choice, "I fought it all this time on my own, and then finally I had to give in and take a pill. That was kind of depressing".

Those diagnosed more recently found it easier to decide to begin treatment, but it was still a significant moment. Tim, diagnosed in 2012, was aware of the latest research and asked to start treatment immediately, even for him, the 'treatment appointment' was a sobering experience:

> *"[It was] the only time there was a tear. I just thought, God this is a new chapter now. This is a new chapter in my life. I am going to have to take this pill for the rest of my life".* (B7, Gen 3 MSM)

Brian, diagnosed in 2013, sums up some of the issues that participants said they had considered when deciding to start medication:

> *"Well the impact it would have on my life, the damage it would do to my body. Would I cope with the medication? Would I be able to continue working? Because so many people have side effects initially and it takes them a long time to get over. I had a lot of responsibility at work and I couldn't actually manage responsibility well enough once on medication. Would I be able to take the medication on time? Would life's pressures allow me to do what I needed to do? And so on"* (B19, Gen 4, MSM*)*

The three participants not on treatment described feeling healthy and wished to remain drug free for as long as possible.

For many participants, HIV occupied only a small part of their lives but daily medications proved to be a constant reminder of their status even if only for a moment each day.

Martin, diagnosed in 2014, described his relationship with his medication:

> "*It's strange sometimes because you look at this pill and you think between you and this little pill lies – it's keeping you alive. And I have never had a pill to take like that before. So, it's very strange. It's my friend and foe at the same time"* (A22, gen 4, MSM)

DISCUSSION

The revolution in HIV treatment over three decades means it can now be described as a chronic, manageable condition.[6, 7] In our study, all our patients were virally supressed having passed through all the stages of the continuum and arrived on the other side. However, patients' recall of

For peer review only - http://bmjopen.bmj.com/site/about/guidelines.xhtml

their experience of navigating this journey revealed a range of quite complex issues faced by them at different points, reminding us that many challenges remain in the successful provision of HIV care. We hypothesized that the revolution in treatment would have an impact on the experience of the different diagnostic generations moving through the care continuum, with those diagnosed more recently having a smoother journey. We identified some important differences; the visible illness and deaths from AIDS made it harder for earlier generations to engage with or remain in care and some dropped out, returning only when ill. Decisions about treatment were particularly difficult for the early generations, for whom the association of treatment with severe side effects remained strong until more recently and who expressed pride in, and determination to, remaining well without treatment.

However, our primary finding was of a striking similarity across participants' accounts of key stages of the HIV care continuum: diagnosis was a major, traumatic life event for almost everyone; anticipation of an HIV-positive status affected the timing of testing and diagnosis; engaging with care was facilitated by a responsive, flexible approach on the part of services and clinicians, starting with how the positive diagnosis was handled; and initiating treatment was a major life decision even when recommended by protocol and considered straightforward by clinicians.

Despite the drive to normalise HIV testing through simplified sampling, reduced pre-test discussion and expanded test settings,[8, 9] [10] receiving an HIV diagnosis remained a significant shock for most participants irrespective of generation, as suggested elsewhere.[11-14] Resonating with other studies.[12, 15, 16] , we found that fear of imminent death and experience of profound distress did not change despite the availability of effective and less toxic treatments. This fear, often coupled with a fear of social exclusion and rejection, led participants who suspected they were positive to delay testing.[17]

A clinician's approach to a patient with a positive result is considered critical to patients' experiences and may be more important than other aspects of the testing process.[11, 14, 18][16, 19] We found negative experiences at this critical point affected immediate well-being and further contact with services. The impact of those initial encounters, both good and bad, left participants with lasting impressions throughout their journey, thus demonstrating the importance of establishing trust between clinician and patient as a firm foundation for good retention in care.[20-22] . Moreover, the importance of that relationship for participants was underlined by their willingness to change their treatment centre until they found what they perceived as a good clinician/patient relationship.

For peer review only - http://bmjopen.bmj.com/site/about/guidelines.xhtml

The UK policy of open access to any clinic through self-referral may be another explanation for high levels of retention in care.

The prospect of treatment for life, to sustain life, was a major life event. Currently the moves towards starting treatment at diagnosis, the test-and-treat model, is based on the confidence of biomedicine in HIV management, but may be at odds with patient concerns.[23] BHIVA interim guidelines 2016 recommend starting treatment on all diagnosed with HIV regardless of CD4 count and continue to recognise that social, psychological, cultural, and economic factors can adversely affect adherence and treatment outcomes.[24] Starting medication on the same day as, or soon after, diagnosis when individuals may be distressed by the positive result could preclude a meaningful discussion of the patient's 'readiness to start'. Persson's 2016 study of patients not on ARVs found similar barriers and concerns: for example, logistics of starting life-long medication, fear of long term side effects and desire to stay drug-free whilst healthy.[25]

The evolution of simpler treatments has been accompanied by a reconfiguration of the care pathway. In London, HIV clinics are increasingly narrowly focused on HIV and HIV-specific medications and clinicians are not authorised to provide more holistic medical care.[26] In practice, this has led to less frequent clinic visits, a shift to virtual "e-clinics", and greater links with, and reliance on, general practitioners. The impact of this on the care continuum is unclear. Continuity of clinician, the atmosphere in the clinic and good communication are recognised to be key issues for patients.[27, 28], were reflected in our findings. It is therefore important that these are not lost with streamlining pathways. All our participants were virally suppressed but that can hide the reality of their reliance on the caregivers to help maintain that stability.

The study's strengths are in the large number of participants, at two different clinics and the inclusion of diverse groups and the four generations. The semi-structured interview format allowed us to explore factors important to participants rather than asking about pre-defined concerns. However, our study sample was imbalanced between generations, with larger numbers of women in the earliest two generation and only one of those more recently diagnosed. This means that some of our conclusions about generation may also reflect gendered differences. Our recruitment methods that relied on recruitment from the two clinics meant we were not able to explore the experiences of those who remain outside care. The study is also limited by the single interview format which meant people were having to recall past experiences, some of which were three decades ago. This may have introduced recall bias with subsequent experience colouring earlier reports; moreover, earlier generations had more time to have disengaged and re-engaged with care.

For peer review only - http://bmjopen.bmj.com/site/about/guidelines.xhtml

A personal, holistic approach has been a hallmark of HIV care since the beginning of the epidemic. It is important that major advances in biomedical treatment do not undermine the care continuum through a loss of care that meets the complex needs of patients, for whom HIV diagnosis and treatment remain significant challenges requiring supportive and flexible care.

Acknowledgements: Thanks to participants for sharing their time and stories, to Jane Rowlands and Chris Higgs for conducting some of the interviews and the staff of both clinics for facilitating the study.

Contributership statement: All four authors, J. Bruton, T. Rai, S. Day and H. Ward, were involved in the design of the study. J. Bruton and T. Rai completed the initial analysis and interpretation of the data and subsequent discussion and analysis was undertaken by all 4 authors through team discussion. J. Bruton completed the first draft of the manuscript. All four authors participated in critiquing and revising the draft manuscript and all approved the final draft.
All four authors accept accountability for the submitted paper.
T. Rai and J. Bruton only undertook the interviews alongside collaborators J. Rowlands and C.Higgs.

Funders: Funded by grants from Imperial NIHR Biomedical Research Centre, P35771, the Imperial College Healthcare Charity, P44223, and supported by the St Stephens AIDS Trust. No pharmaceutical grants were received in the development of this study.

Competing Interests: There are no competing interests

Data sharing statement: The interview transcripts are anonymised and stored securely at Imperial College London on a password protected database on a password protected computer. The participants were assured of the security of their data and they would remain confidential.

For peer review only

References

1. Gardner, E.M., et al., *The Spectrum of Engagement in HIV Care and its Relevance to Test-and-Treat Strategies for Prevention of HIV Infection.* Clinical Infectious Diseases, 2011. **52**(6): p. 793-800.
2. UNAIDS, *UNAIDS. 90-90-90 - an ambitious treatment target to help end the AIDS epidemic (accessed June 21, 2017)* http://www.unaids.org/en/resources/documents/2014/90-90-90. 2014.
3. Kirwan PD, C.C., Brown AE., Gill ON.,Delpech VC. and contributors, *HIV in the UK: 2016 Report*. 2016, Public Health England: London.
4. Flowers, P., et al., *Understanding the impact of HIV diagnosis amongst gay men in Scotland: an interpretative phenomenological analysis.* Psychol Health, 2011. **26**(10): p. 1378-91.
5. Christopoulos, K.A., et al., *"Taking a half day at a time:" patient perspectives and the HIV engagement in care continuum.* AIDS Patient Care STDS, 2013. **27**(4): p. 223-30.
6. Deeks, S.G., S.R. Lewin, and D.V. Havlir, *The End of AIDS: HIV Infection as a Chronic Disease.* Lancet, 2013. **382**(9903): p. 1525-1533.
7. Yehia, B.R., et al., *Structures of Care in the Clinics of the HIV Research Network.* AIDS Patient Care and STDs, 2008. **22**(12): p. 1007-1013.
8. Ogaz D., F.M., Connor N., Gill ON. and contributors, *HIV testing in England:2016 report*. 2016, Public Health England: London England.
9. Rayment, M., et al., *HIV Testing in Non-Traditional Settings – The HINTS Study: A Multi-Centre Observational Study of Feasibility and Acceptability.* PLOS ONE, 2012. **7**(6): p. e39530.
10. Wise, J., *Guidelines call for HIV testing to be "normalised".* BMJ, 2008. **337**.
11. Roth, N.L. and M.S. Nelson, *HIV diagnosis rituals and identity narratives.* AIDS Care, 1997. **9**(2): p. 161-180.
12. Stevens, P.E. and E. Hildebrandt, *Life changing words: women's responses to being diagnosed with HIV infection.* ANS Adv Nurs Sci, 2006. **29**(3): p. 207-21.
13. Stevens, P.E. and B. Tighe Doerr, *Trauma of discovery: women's narratives of being informed they are HIV-infected.* AIDS Care, 1997. **9**(5): p. 523-38.
14. Anderson, M., et al., *"It took a piece of me": initial responses to a positive HIV diagnosis by Caribbean people in the UK.* AIDS Care, 2010. **22**(12): p. 1493-8.
15. Meursing, K. and F. Sibindi, *HIV counselling — a luxury or necessity?* Health Policy and Planning, 2000. **15**(1): p. 17-23.
16. Christopoulos, K.A., et al., *Patient Perspectives on the Experience of Being Newly Diagnosed with HIV in the Emergency Department/Urgent Care Clinic of a Public Hospital.* PLOS ONE, 2013. **8**(8): p. e74199.
17. Clifton, S., et al., *HIV testing, risk perception, and behaviour in the British population.* Aids, 2016. **30**(6): p. 943-52.
18. Worthington, C. and T. Myers, *Desired elements of HIV testing services: test recipient perspectives.* AIDS Patient Care STDS, 2002. **16**(11): p. 537-48.
19. Hult, J.R., S.A. Maurer, and J.T. Moskowitz, *"I'm sorry, you're positive": a qualitative study of individual experiences of testing positive for HIV.* AIDS Care, 2009. **21**(2): p. 185-8.
20. Kempf, M.C., et al., *A qualitative study of the barriers and facilitators to retention-in-care among HIV-positive women in the rural southeastern United States: implications for targeted interventions.* AIDS Patient Care STDS, 2010. **24**(8): p. 515-20.
21. Yehia, B.R., et al., *Barriers and facilitators to patient retention in HIV care.* BMC Infectious Diseases, 2015. **15**(1): p. 246.

For peer review only - http://bmjopen.bmj.com/site/about/guidelines.xhtml

22. Engelhard, E.A., et al., *Impact of HIV care facility characteristics on the cascade of care in HIV-infected patients in the Netherlands.* AIDS, 2016. **30**(2): p. 301-310.
23. Newman, C.E., *Appreciating doubts about HIV medicine*. 2015. Vol. 18. 2015.
24. BHIVA, *British HIV Association guidelines for the treatmnet of HIV-1- positive adults with antiretroviral therapy. 2015 (2016 interim update)*

2016.
25. Persson, A., et al., *On the Margins of Pharmaceutical Citizenship: Not Taking HIV Medication in the "Treatment Revolution" Era.* Med Anthropol Q, 2016. **30**(3): p. 359-77.
26. Miller R., G.J., *Commissioning London's HIV Services*. 2011, Civitas (Institute for the Study of Civil Society).
27. Weatherburn P., K.P., Reid D., Hammond G., Jessup K., *My care I care: A study of what people with HIV value about NHS HIV Services across London*. 2013, Sigma Research.
28. Haggerty, J.L., et al., *Continuity of care: a multidisciplinary review.* BMJ: British Medical Journal, 2003. **327**(7425): p. 1219.

For peer review only

For peer review only - http://bmjopen.bmj.com/site/about/guidelines.xhtml

Key statements

- A qualitative study understanding the patients' perspective of their experience of the HIV care continuum
- The diverse study cohort spans 4 'generations' diagnosed at different time points between 1986 and 2014 reflecting the evolution of ARV's
- There are striking similarities of experience and significance at key points on the continuum: diagnosis, engaging in care and starting treatment
- Despite advances of biomedical treatment clinical services should continue to recognise the needs of patients for whom HIV diagnosis and treatment remains a significant challenge

For peer review only

For peer review only - http://bmjopen.bmj.com/site/about/guidelines.xhtml

Imperial College London

Chelsea and Westminster Hospital NHS
NHS Foundation Trust

Imperial College Healthcare NHS
NHS Trust

HIV patient interview topic guide

Overview description (recap and summary of information in the patient information sheet)

During this project we wish to find out people's own experiences of their life with HIV starting from the beginning, before you were diagnosed and going all the way up to the present. There are some specific areas we wish to cover, however you are free to discuss what you feel are important issues or areas that have been relevant in your HIV journey.

Questions:

[May or may not need to ask all the questions specifically, this is just a guide, some questions overlap]

1. **Personal information – work/relationships/home situation**

   Can you tell me a bit about yourself….
   a. What are your home circumstances?
   b. Are you in a relationship/s?
   c. Do you have dependents children? Parents? partner ?
   d. Do you work? What do you do? Benefits?

2. **Testing decisions**

   a. Can you tell me when you were diagnosed?
   b. How did you come to be tested?
   c. Where did you go for your test? Why?
   d. Had you had HIV tests before, if so why?
   e. What was your health like before your diagnosis?
   f. Had/did you know anybody living with HIV at the time?

3. **Experience of initial care**

   a. What happened when you were given your positive result?
   b. Can you remember what information you were given at the time? Was this given in the way you wanted? Please can you tell me in detail.
   c. What questions, if any, did you have for the clinical staff? Did you ask them?

For peer review only - http://bmjopen.bmj.com/site/about/guidelines.xhtml

d. Did you tell anyone / talk to anyone after you found out your results? Can you tell me a bit more about this – who were they, was it helpful to talk to them, etc.?
e. Did you access information about HIV? How? where? How has this helped?
f. What did you do after receiving your diagnosis?
g. Which HIV clinic did you go to? Why? Are you still at that clinic? Why?

4. Treatment decisions and research

a. Are you on treatment ?
b. How was the decision to start taken? Were you involved in that decision?
c. What preparation did you make?

d. (If not on treatment) What are your thoughts about going on treatment?

(If on treatment) What do you think / have you any thoughts about being on treatment?

e. (If on treatment) How do you find taking your medication?

f. Have you discussed your thoughts about treatment with anyone?
g. Do you know what recommendations there are for when people might start treatment?

h. Are you aware of the research into ARVs and treatment guidelines in the UK?
i. What do you know about them? What do you think of them?
j. How have they influenced your understanding of treatment?
k. Have they influenced your decision to start/change/stop treatment?

l. Do you take part in clinical trials? Why?

5. Clinical care

a. How often do you go to the clinic? Has that changed over the time you have been in the clinic? How?
b. Who do you see? And for what? Doctor? Consultant? Nurse? Peer support? Health Advisor? etc
c. What kind of care have you received at the clinic? Any good/bad experiences?

d. What do you think the role of the clinic is in your care?
e. What other clinical services do you access? Where? How is your care coordinated?
f. What do you think about the way in which HIV services are currently organised? Does it work for you?
g. Have they changed? In what way?
h. Are you aware of possible future changes in the services?

For peer review only - http://bmjopen.bmj.com/site/about/guidelines.xhtml

6. **Primary care /Community and Social care**

   a. Where do you go for your primary care needs? Why? What is your experience of primary care?
   b. How is your care coordinated between primary care and the clinic?
   c. Do you access any social care or voluntary services? If so what? Why?
   d. Do they meet your needs? If not why?
   e. Have you seen any changes in what is available? If so what?

7. **Living with HIV**

   a. Has having HIV affected your life? What are the most significant ways HIV has affected your life, and has that changed over time?
   b. Do you know others living with HIV? What impact does that have on your life?
   c. What kind of support have you had/needed since you were diagnosed positive? Has this changed over time?

8. **Is there anything else you would like to say?**
   a. Given your experience of living with HIV, what would you say to others who have been recently diagnosed about what to expect?

For peer review only

For peer review only - http://bmjopen.bmj.com/site/about/guidelines.xhtml

COREQ (COnsolidated criteria for REporting Qualitative research) Checklist

A checklist of items that should be included in reports of qualitative research. You must report the page number in your manuscript where you consider each of the items listed in this checklist. If you have not included this information, either revise your manuscript accordingly before submitting or note N/A.

Topic	Item No.	Guide Questions/Description	Reported on Page No.
**Domain 1: Research team and reflexivity**			
Personal characteristics			
Interviewer/facilitator	1	Which author/s conducted the interview or focus group?	4
Credentials	2	What were the researcher's credentials? E.g. PhD, MD	1
Occupation	3	What was their occupation at the time of the study?	1
Gender	4	Was the researcher male or female?	1
Experience and training	5	What experience or training did the researcher have?	1
Relationship with participants			
Relationship established	6	Was a relationship established prior to study commencement?	4
Participant knowledge of the interviewer	7	What did the participants know about the researcher? e.g. personal goals, reasons for doing the research	4
Interviewer characteristics	8	What characteristics were reported about the inter viewer/facilitator? e.g. Bias, assumptions, reasons and interests in the research topic	1
**Domain 2: Study design**			
Theoretical framework			
Methodological orientation and Theory	9	What methodological orientation was stated to underpin the study? e.g. grounded theory, discourse analysis, ethnography, phenomenology, content analysis	5
Participant selection			
Sampling	10	How were participants selected? e.g. purposive, convenience, consecutive, snowball	4
Method of approach	11	How were participants approached? e.g. face-to-face, telephone, mail, email	4
Sample size	12	How many participants were in the study?	5
Non-participation	13	How many people refused to participate or dropped out? Reasons?	NA
Setting			
Setting of data collection	14	Where was the data collected? e.g. home, clinic, workplace	4
Presence of non-participants	15	Was anyone else present besides the participants and researchers?	4
Description of sample	16	What are the important characteristics of the sample? e.g. demographic data, date	5/6
Data collection			
Interview guide	17	Were questions, prompts, guides provided by the authors? Was it pilot tested?	5
Repeat interviews	18	Were repeat inter views carried out? If yes, how many?	NA
Audio/visual recording	19	Did the research use audio or visual recording to collect the data?	4
Field notes	20	Were field notes made during and/or after the inter view or focus group?	5
Duration	21	What was the duration of the inter views or focus group?	4
Data saturation	22	Was data saturation discussed?	N/A
Transcripts returned	23	Were transcripts returned to participants for comment and/or	NA

For peer review only - http://bmjopen.bmj.com/site/about/guidelines.xhtml

Topic	Item No.	Guide Questions/Description	Reported on Page No.
		correction?	
**Domain 3: analysis and findings**			
Data analysis			
Number of data coders	24	How many data coders coded the data?	5
Description of the coding tree	25	Did authors provide a description of the coding tree?	NA
Derivation of themes	26	Were themes identified in advance or derived from the data?	5
Software	27	What software, if applicable, was used to manage the data?	5
Participant checking	28	Did participants provide feedback on the findings?	NA
Reporting			
Quotations presented	29	Were participant quotations presented to illustrate the themes/findings? Was each quotation identified? e.g. participant number	6,7,8,9
Data and findings consistent	30	Was there consistency between the data presented and the findings?	9,10,11
Clarity of major themes	31	Were major themes clearly presented in the findings?	9,10,11
Clarity of minor themes	32	Is there a description of diverse cases or discussion of minor themes?	9,10,11

Developed from: Tong A, Sainsbury P, Craig J. Consolidated criteria for reporting qualitative research (COREQ): a 32-item checklist for interviews and focus groups. *International Journal for Quality in Health Care*. 2007. Volume 19, Number 6: pp. 349 – 357

Once you have completed this checklist, please save a copy and upload it as part of your submission. DO NOT include this checklist as part of the main manuscript document. It must be uploaded as a separate file.

For peer review only

Patient perspectives on the HIV continuum of care in London: a qualitative study of people diagnosed between 1986 and 2014

Journal:	*BMJ Open*
Manuscript ID	bmjopen-2017-020208.R1
Article Type:	Research
Date Submitted by the Author:	05-Jan-2018
Complete List of Authors:	Bruton, Jane; Imperial College Lodon, Department of Infectious Disease Epidemiology<br>Rai, Tanvi; Imperial College Lodon, Department of Infectious Disease Epidemiology<br>Day, Sophie; Imperial College Lodon, Department of Infectious Disease Epidemiology; Goldsmiths University of London, Anthropology<br>Ward, Helen; Imperial College London, Department of Infectious Disease Epidemiology
**Primary Subject Heading**:	HIV/AIDS
Secondary Subject Heading:	Qualitative research, Health services research, Patient-centred medicine
Keywords:	HIV & AIDS < INFECTIOUS DISEASES, QUALITATIVE RESEARCH, PATIENT EXPERIENCE, HIV CARE CONTINUUM

SCHOLARONE™ Manuscripts

Patient perspectives on the HIV continuum of care in London: a qualitative study of people diagnosed between 1986 and 2014

Jane Bruton RN, BA, MA Medical Anthropology; Tanvi Rai PhD Public Health, MPH, BA, BSc; Sophie Day, PhD, BA, MA Anthropology; BSc Helen Ward PhD, FRCP, FFPH, MBChB, MSc Epidemiology.

Corresponding author:
Pamela Jane Bruton,
Department of Infectious Disease Epidemiology,
School of Public Health,
Imperial College,
St Mary's Campus,
Norfolk Place,
London.
W2 1PG
jbruton@ic.ac.uk
0207 5943464

Co-authors:
Rai, Tanvi: Imperial College London, Department of Infectious Disease Epidemiology.
Day, Sophie: Imperial College London, Department of Infectious Disease Epidemiology and Goldsmiths College
Ward, Helen: Imperial College London, Department of Infectious Disease Epidemiology.

Word count: Main text 3410

For peer review only

For peer review only - http://bmjopen.bmj.com/site/about/guidelines.xhtml

Abstract

Background: The care continuum provides a step-wise measure of success in managing HIV. Whilst useful for providers it does not inform us of the actual experience of individuals negotiating the different stages of the HIV care pathway. Recognising the revolution in HIV treatments, the consequent transformation from an acute life-threatening to a chronic long-term condition, and changes in care delivery, we hypothesised that patients' experience may differ according the point at which they were diagnosed in the epidemic.

Methods: Qualitative interview study of 52 individuals attending two large London HIV clinics, purposively sampled, on the basis of being diagnosed at different stages in the history of the epidemic, into HIV "generations" and analysed thematically.

Results: Some important differences were identified; for earlier generations, the visible illness and deaths from AIDS made it harder to engage with care following diagnosis. Subsequent decisions about starting treatment were deeply influenced by the fear of severe side-effects from early antiretroviral therapy (ART). However, despite improvements in ART and life expectancy over the epidemic we found a striking similarity across participants' accounts of the key stages of the care continuum, regardless of when they were diagnosed. Diagnosis was a major traumatic life event for almost everyone, fear of testing positive or having low self-perceived risk affected the timing of testing and diagnosis, engaging with care was facilitated by a flexible approach from services/clinicians and initiating treatment was a major life decision.

Conclusion: We found patients' experiences are influenced by when they were diagnosed, with earliest cohorts facing substantial challenges. However, being diagnosed with HIV and starting treatment continue to be significant life-altering events even in the era of effective, simple treatments. Despite the advances of biomedical treatment services should continue to recognise the needs of patients for whom the diagnosis and treatment remain significant challenges.

For peer review only

For peer review only - http://bmjopen.bmj.com/site/about/guidelines.xhtml

Strengths

- The large number of participants, at two different clinics and the inclusion of a range of people with HIV, broadly similar to the clinic cohorts, across four HIV 'generations'.
- The interview format allowed us to explore factors important to participants rather than asking about pre-defined concerns.

Limitations

- An imbalance between generations, with larger numbers of women in the earliest two generations and only one woman in the generation recently diagnosed. This means that some of our conclusions about generations may also reflect gendered differences.
- Our recruitment methods meant we were not able to explore the experiences of those who remain outside care.
- Limitation of the single interview format means people were having to recall past experiences, some of which were three decades ago. This may have introduced recall bias with subsequent experience colouring earlier reports.

For peer review only

For peer review only - http://bmjopen.bmj.com/site/about/guidelines.xhtml

INTRODUCTION

The Human Immunodeficiency Virus (HIV) continuum of care provides stepwise estimates for the stages of engagement in care for people living with HIV (PLHIV).(1) The Joint United Nations Programme on HIV and AIDS (UNAIDS) goal to end the Acquired Immune Deficiency Syndrome (AIDS) epidemic by 2020 stipulates a target of 90% of all people with HIV be diagnosed, of whom 90% are on antiretroviral therapy (ART), of whom 90% (73% of PLHIV) are virally suppressed.(2) The United Kingdom (UK) has one of the best outcomes for HIV treatment and care in the world with an estimated 78% of PLHIV having an undetectable viral load.(3, 4) However the statistics do not and cannot tell us about the patient experience of passing through these stages of care whether it be good or bad. The continuum essentially measures the success of programmes from a provider rather than a patient perspective.(5)

Flowers argues that there is a tension between the certainty and confidence of a linear HIV pathway, associated with ideas of clinical efficacy, and patient experiences of diagnosis and prognosis, which can be full of uncertainty.(4) With the evolution of modern ARTs we have witnessed the transformation of HIV from an acute life-threatening infection to a treatable chronic condition and a concurrent evolving of the care continuum. However, it is unclear whether this change is reflected in patients' own experiences of passing through each of the stages of care. For example, has the moment of diagnosis become any less traumatic, and have decisions about starting treatment become simpler for patients? Analysis of patient narratives, historically and currently, may help to highlight significant factors for patients in the care continuum. This is particularly important in a climate of National Health Service (NHS) restructuring in the UK, which has led to reductions in non-clinical services and streamlining of care. To this end we explored the patient perspectives on the care continuum. And we hypothesized that patients' experiences of, and engagement with, care would differ according to what point in the epidemic they were diagnosed.

For peer review only

METHODS

We undertook a qualitative study of people attending two public HIV clinics in London that have provided care since the start of the epidemic; they were also chosen for their large size and diversity, in terms of demographics. They are both specialist HIV clinics linked to sexual health (genitourinary medicine) services. Care is provided by physician-led multidisciplinary teams where patients have a named consultant. HIV care in the UK is free and open access, allowing patients to register at their clinic of choice. We used a purposive sampling method to recruit patients with a range of experiences. To reflect the evolution of antiretroviral therapy (ART), we identified four 'generations' according to time of diagnosis: pre-1996 (pre-ART), 1997 to 2005 (complex ARTs), 2006 to 2012

For peer review only - http://bmjopen.bmj.com/site/about/guidelines.xhtml

(simpler ARTs), 2013 onwards (recent diagnoses). Within each generation, we aimed to include people with a range of characteristics, including, gender, exposure, age and ethnicity. Participants were recruited opportunistically by researchers attending clinical services, and through fliers and digital advertising in clinical areas. Recruitment was periodically checked against the recruitment matrix and under-represented groups/strata targeted.

Patients were provided with information and gave written consent. Interviews took place in private rooms in or near the clinics or at the patient's home; they were recorded and transcribed, and lasted between 60 and 90 minutes. The interviews were semi-structured and carried out by one male and three female researchers (JB, TR, CH, JR), three of whom had clinical backgrounds. The interviews were based on a topic guide (appended) informed by a focus group of PLHIV who assisted in designing the research. We invited participants to recall their initial diagnosis and describe key points in their HIV journey including testing, disclosure, support, engaging with care, starting treatment, medication adherence, work and social life. Field notes were written after the interviews.

Transcripts were uploaded to NVivo, a qualitative data analysis software package. Using Framework analysis, we developed key themes through a systematic process which involved reading, rereading, coding and summarising the transcripts; and subsequent in-depth analysis of the dataset.(6)Final themes were discussed in the research group (HW, JB, SD, TR) and further analysed in relation to the existing literature.

Ethical approval was obtained from the National Research Ethics Service (NRES) (reference number 14/WM/0147) in May 2014, and research governance approval obtained from the local sites.

RESULTS

Fifty-two patients were recruited, 25 at one clinic and 27 at the other. The sample included 41 men and 11 women; 37 men acquired HIV through sex with other men (MSM), the rest through heterosexual contact (14) or injection drug use (1). There were 11 in generation 1, 14 generation 2, 17 generation 3 and 10 generation 4. The characteristics of the study participants alongside those of the clinic population for 2014 are shown in Table 1.

Table 1. Study sample characteristics compared with the clinic cohorts

	Clinic A				Clinic B			
	Study sample		2014 Cohort		Study sample		2014 Cohort	
**Gender**								
Male	18	72.0%	2459	77.5%	23	85.2%	7743	90.3%
Female	7	28.0%	715	22.5%	4	14.8%	830	9.7%

For peer review only - http://bmjopen.bmj.com/site/about/guidelines.xhtml

**Age**								
18-24	0	0.0%	124	3.9%	0	0.0%	186	2.2%
25-34	4	16.0%	465	14.7%	3	11.1%	1729	20.2%
35-49	16	64.0%	1502	47.3%	14	51.9%	4164	48.6%
50+	5	20.0%	1083	34.1%	10	37.0%	2494	29.1%
**Ethnicity**								
White	14	56.0%	1541	48.6%	22	81.5%	6401	74.7%
Black African	5	20.0%	720	22.7%	5	18.5%	778	9.1%
Black Caribbean	0	0.0%	114	3.6%	0	0.0%	201	2.3%
Other/Mixed	6	24.0%	746	23.5%	0	0.0%	1132	13.2%
Not reported	0	0.0%	53	1.7%	0	0.0%	61	0.7%
**Exposure route**								
Sex between men	15	60.0%	1971	62.1%	22	81.5%	6776	79.0%
Heterosexual contact	9	36.0%	1037	32.7%	5	18.5%	1209	14.1%
Injecting drug use	1	4.0%	52	1.6%	0	0.0%	109	1.3%
Other	0	0.0%	112	3.5%	0	0.0%	46	0.5%
Undetermined	0	0.0%	2	0.1%	0	0.0%	433	5.1%
**Year of diagnosis**								
Pre-1997	6	24.0%	638	20.1%	5	18.5%	1399	16.3%
1997-2005	6	24.0%	1234	38.9%	8	29.6%	2580	30.1%
2006-2012	7	28.0%	986	31.1%	10	37.0%	3199	37.3%
2013 onwards	6	24.0%	316	10.0%	4	14.8%	1395	16.3%
Total			3174				8573	

The generation samples differed somewhat by gender and acquisition: the women were concentrated in generations 1 and 2 (six and four respectively), and MSM in generations 3 and 4 (16 and eight).

"Becoming positive" – the impact of HIV diagnosis

The experience of receiving an HIV diagnosis was similar across the generations. First reactions were generally of shock and fear of death, irrespective of generation. Alan (pseudonym), diagnosed in 1991, knew nothing about HIV and had not tested before. He remembers vividly the time he received his result:

> *"I could hear myself saying 'I'm going to die'. Not verbally but in my mind, 'I'm going to die, I'm going to die'"* (gen1, MSM)

For peer review only

For peer review only - http://bmjopen.bmj.com/site/about/guidelines.xhtml

Sylvia, diagnosed 2001, was “totally devastated”:

> *“… I didn’t see myself going back and doing my Master’s degree for what reason am I going back to do that if I have maybe five years to live”* (gen 2, woman)

Roger, diagnosed more than 20 years later, reported several previous tests and considered himself well-informed. However, his principal concern on receiving a positive diagnosis was also about life expectancy:

> *“But even I was not certain. Certainty is the wrong word. I was under the illusion that my expiry date was stamped on me now”* (gen4, MSM)

Fear of a positive result was a key factor in delayed diagnosis for several MSM in all generations, who reported concerns about the impact of HIV on their lives. They were aware of their risk, and described feeling relieved at diagnosis as HIV had been *“hanging over them”* for years; the diagnosis confirmed their suspicions. William, who had never tested before, presented with symptoms:

> *“[I had been] burying my head in the sand. I guess I knew I had it but didn’t, at the same time, want it confirmed”* (gen2, MSM)

Brian (Gen4, MSM), recently diagnosed, had *“spent on and off probably eight years thinking about it*” He felt he had “*done all the thinking before*” … so although disappointed, he was also relieved.

Most other participants, particularly heterosexual men and women, were not expecting a positive result and had not requested an HIV test. They were diagnosed either following ongoing symptoms of ill health or having presented for a general sexual health check-up.

None of the women were diagnosed through routine ante-natal screening. For example, Olivia, diagnosed in 1998, had not been tested in pregnancy. Her six-month old baby became sick, and both baby and husband were then diagnosed with HIV but she did not believe she had HIV and delayed testing for several weeks

> *“Me I don’t have HIV because I never went with other men”* (gen2, woman)

The response and level of support offered by clinicians at this critical time were important to participants’ immediate wellbeing and influenced what happened next, including continuing engagement in care. Martha remarked:

> *“I remember how lovely [name of clinician] was and I’ve always said I could never wish for a better person to ever tell me or try to guide me, or to reassure me more than what she did because she was perfect”* (Gen4, woman)

While most experiences were positive, there were some exceptions. Paul (gen3, MSM) had regularly tested negative but continued to take risks. Testing positive in 2010 was totally unexpected, leaving

For peer review only - http://bmjopen.bmj.com/site/about/guidelines.xhtml

him “*numb with shock*”, and he did not feel that he was supported appropriately. The clinician who gave him the diagnosis seemed “[to be on] *autopilot because he had seen people like me before*” and was “*working to his own agenda*”. Despite Paul’s obvious distress, the clinician asked him to ring potential contacts during the consultation. Further, when the clinician said: “*Oh this can be managed, don’t worry*”, Paul interpreted this to mean ‘managed to his death’. After two weeks of acute anxiety, Paul contacted a friend who was able to reassure him about treatment and the care pathway. Similar experiences led other participants to feel vulnerable, isolated and slow to accept their diagnosis.

“Becoming an HIV patient”: Developing a relationship with clinic and clinician

Once diagnosed, participants described a sense of reassurance about being in the “best hands*”,* managed by experts in HIV medicine. The majority across the generations described strong relationships with their clinicians and valued seeing the same person each visit. It felt “*like a partnership*” with “*someone you can tell anything*”, who knew them and their entire history, ensuring that care went beyond just the clinical management of HIV: “*We seriously talk about how I am not just what my CD4 count is*”.

However, some had not developed a trusting relationship. Marty (gen3, MSM), for example, diagnosed HIV in 2012, was not eligible for treatment under guidelines at that point. He described anxiety about this lack of treatment, feeling that it adversely affected pre-existing mental health problems which were not addressed by his clinicians. He attended two different clinics and was on the verge of dropping out of care when, as he recounted: *“I basically rescued myself*”. His friend recommended a clinician: “*She got me just like that thank god, thank god…I finally found and she was willing to fight my case*”.

Another participant, Peter, diagnosed 2009, reported changing HIV clinics within three months of diagnosis. He recalled a series of mistakes, miscommunication and a “dehumanising” clinic environment. Losing trust in clinicians and the service, he finally gained confidence from attending a support group and moved his care:

> “*I remember that I said, it was like falling off a building… I’m slowly falling backwards as the virus increases. It felt like they were holding a blanket at the bottom to catch me but it felt like they were holding it in the wrong place. I was being asked to trust”* (gen3, MSM)

For peer review only - http://bmjopen.bmj.com/site/about/guidelines.xhtml

All participants valued continuity of care although two recently diagnosed felt that it was not always necessary to see the same clinician. But, continuity was affected by what some described as the very busy clinics limiting the time for consultations and impeding communication.

All participants were in care at the time of the interview but some described having stayed away in the past. Two of six women diagnosed before 1996 had dropped out of care for several years. Given the lack of effective treatment, they had found clinic visits depressing and preferred to keep away until they became sick. Marie (gen1, woman) explained, *"I didn't want a life where I just would go to tests and I am scared and they had nothing to offer".* Alison (gen1, woman) described the *"terrible situation"* at the clinic, where she saw young gay men, couples, where one would be fit and the other *"in a wheelchair, a skeleton".* She felt sorry for the doctors - *"there were all these poor young doctors with nothing to offer and seeing these very ill people."*

Seven of all those diagnosed since ARTs became available described occasional or multiple lapses in attendance; these were generally explained by issues external to the clinic such as recreational drug use, household disruption, mental health problems or competing co-morbidities. Re-engagement with care was easier when their clinician actively reached out; for example, some consultants had telephoned patients when they missed appointments and one woman (gen1) described interventions of this kind as being "*like my family*". Even though not all patients were contacted when they did not attend services, and one expressed surprise that no-one had tried, all found their way back into care.

"Becoming medicalised": Starting treatment

Almost all (48) of the participants were on treatment; one had stopped medication because of drug interactions and three chose to remain off treatment. Despite the simplification of regimens, participants in all four generations found the decision to start treatment a significant life event.

For earlier generations, the decision had been complex because of toxicity related to ARTs. Some refused treatment contrary to medical advice. Alison, described earlier, recalled, "*In the waiting rooms people said, 'don't take it, it will kill you', so I refused that (AZT*)". Others felt well without treatment like Marie who was diagnosed in 1986 but only started ARTs 25 years after diagnosis when her CD4 count crashed. She felt she had no choice, "I fought it all this time on my own, and then finally I had to give in and take a pill. That was kind of depressing".

For peer review only - http://bmjopen.bmj.com/site/about/guidelines.xhtml

Those diagnosed more recently found it easier to decide to begin treatment, but it was still a significant moment. Tim, diagnosed in 2012, was aware of the latest research and asked to start treatment immediately, even for him, the ‘treatment appointment’ was a sobering experience:

> *"[It was] the only time there was a tear. I just thought, God this is a new chapter now. This is a new chapter in my life. I am going to have to take this pill for the rest of my life".* (, gen 3 MSM)

Brian, diagnosed in 2013, sums up some of the issues that participants said they had considered when deciding to start medication:

> *"Well the impact it would have on my life, the damage it would do to my body. Would I cope with the medication? Would I be able to continue working? Because so many people have side effects initially and it takes them a long time to get over. I had a lot of responsibility at work and I couldn't actually manage responsibility well enough once on medication. Would I be able to take the medication on time? Would life's pressures allow me to do what I needed to do? And so on"* (gen 4, MSM)

The three participants not on treatment described feeling healthy and wished to remain drug free for as long as possible.

For many participants, HIV occupied only a small part of their lives but daily medications proved to be a constant reminder of their status even if only for a moment each day. Martin, diagnosed in 2014, described his relationship with his medication:

> *"It's strange sometimes because you look at this pill and you think between you and this little pill lies – it's keeping you alive. And I have never had a pill to take like that before. So, it's very strange. It's my friend and foe at the same time"* (gen 4, MSM)

DISCUSSION

We have found that patients' experiences of, and engagement with, care are influenced by the point at which they were diagnosed, with the earliest cohorts facing substantial challenges. However, we have also found that being diagnosed with HIV and starting treatment continue to be significant life-altering events even in the era of effective and simple treatments. This study brings new insights which are important when considering how future services should be provided.

The revolution in HIV treatment over three decades means it can now be described as a chronic, manageable condition.(7, 8) In our study, all our patients were virally supressed having passed

For peer review only - http://bmjopen.bmj.com/site/about/guidelines.xhtml

through all the stages of the continuum and arrived on the other side. However, patients' recall of their experience of navigating this journey revealed a range of quite complex issues faced by them at different points, reminding us that many challenges remain in the successful provision of HIV care. We hypothesized that the revolution in treatment would have an impact on the experience of the different diagnostic generations moving through the care continuum, with those diagnosed more recently having a smoother journey. We identified some important differences; the visible illness and deaths from AIDS made it harder for earlier generations to engage with or remain in care and some dropped out, returning only when ill. Decisions about treatment were particularly difficult for the early generations, for whom the association of treatment with severe side effects remained strong until more recently and who expressed pride in, and determination to, remaining well without treatment.

However, our primary finding was a striking similarity across participants' accounts of key stages of the HIV care continuum: diagnosis was a major, traumatic life event for almost everyone, and anticipation of an HIV-positive status affected the timing of testing and diagnosis. Engagement with care was facilitated by a responsive, flexible approach on the part of services and clinicians, starting with the way the positive diagnosis was handled. Finally, initiating treatment was a major life decision even when recommended by protocol and considered straightforward by clinicians.

Despite the drive to normalise HIV testing through simplified sampling, reduced pre-test discussion and expanded test settings,(9, 10) (11) receiving an HIV diagnosis remained a significant shock for most participants irrespective of generation, sexuality or gender, as suggested elsewhere.(12-15) Moreover, we found that none of the heterosexual participants were expecting a positive result, a finding which also applied to some of the MSM participants. Resonating with other studies.(13, 16, 17) , we found that fear of imminent death and experience of profound distress did not change, despite the availability of effective and less toxic treatments. This fear, often coupled with a fear of social exclusion and rejection, led some MSM participants who suspected they were positive to delay testing.(18) Bury (1982) usefully describes this experience of illness and especially chronic illness as "biographical disruption". When everyday life and its meanings are turned upside down, relationships and social networks are disrupted and plans for the future have to be re-examined. (19) Participants described this type of disruption at diagnosis, whether HIV was considered an acute infection or a chronic condition.

A clinician's approach to a patient with a positive result is considered critical to patients' experiences and may be more important than other aspects of the testing process.(12, 15, 17, 20, 21) We found that negative experiences at this critical point affected immediate well-being and further contact

For peer review only - http://bmjopen.bmj.com/site/about/guidelines.xhtml

with services. The impact of those initial encounters, both good and bad, left participants with lasting impressions throughout their journey, thus demonstrating the importance of establishing trust between clinician and patient as a firm foundation for good retention in care.(22-24) Women, a minority in the clinics and, in our sample mainly from the earlier generations, faced particular challenges in engaging in care. This made the establishment of a trusting relationship with their clinician all the more important to managing their quality of life with HIV. Moreover, the importance of that relationship for participants was underlined by their willingness to change their treatment centre until they found what they perceived as a good clinician/patient relationship. The UK policy of open access to any clinic through self-referral may be another explanation for high levels of retention in care.

The prospect of treatment for life, to sustain life, was a major life event. Currently the moves towards starting treatment at diagnosis, the test-and-treat model, is based on the confidence of biomedicine in HIV management, but may be at odds with patient concerns.(25) British HIV Association (BHIVA) interim guidelines 2016 recommend starting treatment on all diagnosed with HIV regardless of CD4 count and continue to recognise that social, psychological, cultural, and economic factors can adversely affect adherence and treatment outcomes.(26) Starting medication on the same day as, or soon after, diagnosis when individuals may be distressed by the positive result could preclude a meaningful discussion of the patient's 'readiness to start'. Persson's 2016 study of patients not on ARTs found similar barriers and concerns: for example, logistics of starting life-long medication, fear of long term side effects and desire to stay drug-free whilst healthy.(27)

Considering ARTs was another point at which some participants anticipated 'biographical disruption' which deterred them from starting treatment.

The study's strengths are in the large number of participants at two different clinics, the inclusion of a range of people with HIV broadly similar to the cohorts seen at these clinics, and diagnosed across the four generations. The semi-structured interview format allowed us to explore factors important to participants rather than asking about pre-defined concerns. However, our study sample was imbalanced between generations, with larger numbers of women in the earliest two generation and only one of those more recently diagnosed. This means that some of our conclusions about generation may also reflect gendered differences. Our recruitment methods that relied on recruitment from the two clinics meant we were not able to explore the experiences of those who remain outside care. The study is also limited by the single interview format which meant people were having to recall past experiences, some of which were three decades ago. This may have introduced recall bias with subsequent experience colouring earlier reports; moreover, earlier

For peer review only - http://bmjopen.bmj.com/site/about/guidelines.xhtml

generations had more time to have disengaged and re-engaged with care. The focus of the study on a particular model of care in London limits the generalisability of our findings to other settings.

The evolution of simpler treatments has been accompanied by a reconfiguration of the care pathway. In London, HIV clinics are increasingly narrowly focused on HIV and HIV-specific medications and clinicians are not authorised to provide more holistic medical care.(28) In practice, this has led to less frequent clinic visits, a shift to virtual "e-clinics", and greater links with, and reliance on, general practitioners. The impact of this on the care continuum is unclear. Continuity of clinician, the atmosphere in the clinic and good communication are recognised to be key issues for patients. (29, 30)

There is increasing recognition that viral suppression is not the final goal for people who are now living longer with HIV.(31-33) Lazarus et al (2016) have called for a 'fourth 90' "providing an explicit target for health- related quality of life"(31). In December2017 these concerns were embodied in policy recommendations from the European Parliament calling for an integrated and patient-centred approach to long term HIV care ensuring that services are meeting this challenge. (34) Our study illustrates that the patient journey is complex and personalised care should not be lost with streamlining pathways. All our participants were virally suppressed but that can hide the reality of their reliance on the caregivers to help maintain that stability.

A personal, holistic approach has been a hallmark of HIV care since the beginning of the epidemic. It is important that major advances in biomedical treatment do not undermine the care continuum through a loss of care that meets the complex needs of patients, for whom HIV diagnosis and treatment remain significant challenges requiring supportive and flexible care.

For peer review only

For peer review only - http://bmjopen.bmj.com/site/about/guidelines.xhtml

Acknowledgements: Thanks to participants for sharing their time and stories, to Jane Rowlands and Chris Higgs for conducting some of the interviews and the staff of both clinics for facilitating the study.

Contributership statement: All four authors, J. Bruton, T. Rai, S. Day and H. Ward, were involved in the design of the study. J. Bruton and T. Rai completed the initial analysis and interpretation of the data and subsequent discussion and analysis was undertaken by all 4 authors through team discussion. J. Bruton completed the first draft of the manuscript. All four authors participated in critiquing and revising the draft manuscript and all approved the final draft.
All four authors accept accountability for the submitted paper.
T. Rai and J. Bruton only undertook the interviews alongside collaborators J. Rowlands and C.Higgs.

Funders: Funded by grants from Imperial NIHR Biomedical Research Centre, P35771, the Imperial College Healthcare Charity, P44223, and supported by the St Stephens AIDS Trust. No pharmaceutical grants were received in the development of this study.

Competing Interests: There are no competing interests

Data sharing statement: The interview transcripts are anonymised and stored securely at Imperial College London on a password protected database on a password protected computer. The participants were assured of the security of their data and they would remain confidential.

References

1. Gardner EM, McLees MP, Steiner JF, del Rio C, Burman WJ. The Spectrum of Engagement in HIV Care and its Relevance to Test-and-Treat Strategies for Prevention of HIV Infection. Clinical Infectious Diseases. 2011;52(6):793-800.
2. UNAIDS. UNAIDS. 90-90-90 - an ambitious treatment target to help end the AIDS epidemic (accessed June 21, 2017) http://www.unaids.org/en/resources/documents/2014/90-90-90. 2014.
3. Kirwan PD CC, Brown AE., Gill ON.,Delpech VC. and contributors. HIV in the UK: 2016 Report. London: Public Health England; 2016.
4. Flowers P, Davis MM, Larkin M, Church S, Marriott C. Understanding the impact of HIV diagnosis amongst gay men in Scotland: an interpretative phenomenological analysis. Psychology & health. 2011;26(10):1378-91.
5. Christopoulos KA, Massey AD, Lopez AM, Geng EH, Johnson MO, Pilcher CD, et al. "Taking a half day at a time:" patient perspectives and the HIV engagement in care continuum. AIDS patient care and STDs. 2013;27(4):223-30.
6. Gale NK, Heath G, Cameron E, Rashid S, Redwood S. Using the framework method for the analysis of qualitative data in multi-disciplinary health research. BMC Medical Research Methodology. 2013;13(1):117.

For peer review only - http://bmjopen.bmj.com/site/about/guidelines.xhtml

For peer review only

7. Deeks SG, Lewin SR, Havlir DV. The End of AIDS: HIV Infection as a Chronic Disease. Lancet. 2013;382(9903):1525-33.
8. Yehia BR, Gebo KA, Hicks PB, Korthuis PT, Moore RD, Ridore M, et al. Structures of Care in the Clinics of the HIV Research Network. AIDS patient care and STDs. 2008;22(12):1007-13.
9. Ogaz D. FM, Connor N., Gill ON. and contributors. HIV testing in England:2016 report. London England: Public Health England; 2016.
10. Rayment M, Thornton A, Mandalia S, Elam G, Atkins M, Jones R, et al. HIV Testing in Non-Traditional Settings – The HINTS Study: A Multi-Centre Observational Study of Feasibility and Acceptability. PLOS ONE. 2012;7(6):e39530.
11. Wise J. Guidelines call for HIV testing to be "normalised". BMJ. 2008;337.
12. Roth NL, Nelson MS. HIV diagnosis rituals and identity narratives. AIDS Care. 1997;9(2):161-80.
13. Stevens PE, Hildebrandt E. Life changing words: women's responses to being diagnosed with HIV infection. ANS Advances in nursing science. 2006;29(3):207-21.
14. Stevens PE, Tighe Doerr B. Trauma of discovery: women's narratives of being informed they are HIV-infected. AIDS Care. 1997;9(5):523-38.
15. Anderson M, Elam G, Gerver S, Solarin I, Fenton K, Easterbrook P. "It took a piece of me": initial responses to a positive HIV diagnosis by Caribbean people in the UK. AIDS Care. 2010;22(12):1493-8.
16. Meursing K, Sibindi F. HIV counselling — a luxury or necessity? Health Policy and Planning. 2000;15(1):17-23.
17. Christopoulos KA, Massey AD, Lopez AM, Hare CB, Johnson MO, Pilcher CD, et al. Patient Perspectives on the Experience of Being Newly Diagnosed with HIV in the Emergency Department/Urgent Care Clinic of a Public Hospital. PLOS ONE. 2013;8(8):e74199.
18. Clifton S, Nardone A, Field N, Mercer CH, Tanton C, Macdowall W, et al. HIV testing, risk perception, and behaviour in the British population. AIDS (London, England). 2016;30(6):943-52.
19. Bury M. Chronic illness as biographical disruption. Sociology of health & illness. 1982;4(2):167-82.
20. Worthington C, Myers T. Desired elements of HIV testing services: test recipient perspectives. AIDS patient care and STDs. 2002;16(11):537-48.
21. Hult JR, Maurer SA, Moskowitz JT. "I'm sorry, you're positive": a qualitative study of individual experiences of testing positive for HIV. AIDS Care. 2009;21(2):185-8.
22. Kempf MC, McLeod J, Boehme AK, Walcott MW, Wright L, Seal P, et al. A qualitative study of the barriers and facilitators to retention-in-care among HIV-positive women in the rural southeastern United States: implications for targeted interventions. AIDS patient care and STDs. 2010;24(8):515-20.
23. Yehia BR, Stewart L, Momplaisir F, Mody A, Holtzman CW, Jacobs LM, et al. Barriers and facilitators to patient retention in HIV care. BMC Infectious Diseases. 2015;15(1):246.
24. Engelhard EA, Smit C, Van Sighem A, Reiss P, Nieuwkerk PT, Kroon FP, et al. Impact of HIV care facility characteristics on the cascade of care in HIV-infected patients in the Netherlands. AIDS (London, England). 2016;30(2):301-10.
25. Newman CE. Appreciating doubts about HIV medicine2015.
26. BHIVA. British HIV Association guidelines for the treatmnet of HIV-1- positive adults with antiretroviral therapy. 2015 (2016 interim update) 2016.

27. Persson A, Newman CE, Mao L, de Wit J. On the Margins of Pharmaceutical Citizenship: Not Taking HIV Medication in the "Treatment Revolution" Era. Medical anthropology quarterly. 2016;30(3):359-77.
28. Miller R. GJ. Commissioning London's HIV Services. Civitas (Institute for the Study of Civil Society); 2011.
29. Weatherburn P. KP, Reid D., Hammond G., Jessup K. My care I care: A study of what people with HIV value about NHS HIV Services across London. Sigma Research; 2013.

For peer review only - http://bmjopen.bmj.com/site/about/guidelines.xhtml

30. Haggerty JL, Reid RJ, Freeman GK, Starfield BH, Adair CE, McKendry R. Continuity of care: a multidisciplinary review. BMJ: British Medical Journal. 2003;327(7425):1219.
31. Lazarus JV, Safreed-Harmon K, Barton SE, Costagliola D, Dedes N, del Amo Valero J, et al. Beyond viral suppression of HIV – the new quality of life frontier. BMC Medicine. 2016;14(1):94.
32. Highlights from the BHIVA Satellite Symposium, IAS Conference, Paris, France, July 2017: 'Tougher times: adapting to increasing demand with declining resources'. Journal of Virus Eradication. 2017;3(4):250-2.
33. Martel K CJ, Auberbach J. Looking Beyond Viral Suppression: Findings from The Well Project's User Survey on Factors Influeincing the Health, Well-being, and Quality of Life of Women Living with HIV [IAS 2017 Poster]. 2017 [Available from: http://www.thewellproject.org/news-press/looking-beyond-viral-suppression-findings-well-project%E2%80%99s-2016-user-survey-factors.
34. HIV Outcomes, Beyond Viral Suppression. Recommendations Launched at the European Parliament. 2017 [cited 03/01/2018]. [cited 03/01/2018]. Available from: http://hivoutcomes.eu/wp-content/uploads/2017/11/HIV_Booklet_FINAL-DIGITAL-version.pdf.

For peer review only

For peer review only - http://bmjopen.bmj.com/site/about/guidelines.xhtml

Key statements

- A qualitative study understanding the patients' perspective of their experience of the HIV care continuum
- The diverse study cohort spans 4 'generations' diagnosed at different time points between 1986 and 2014 reflecting the evolution of ARTs
- There are striking similarities of experience and significance at key points on the continuum: diagnosis, engaging in care and starting treatment
- Despite advances of biomedical treatment clinical services should continue to recognise the needs of patients for whom HIV diagnosis and treatment remains a significant challenge

For peer review only

For peer review only - http://bmjopen.bmj.com/site/about/guidelines.xhtml

Imperial College London

Chelsea and Westminster Hospital NHS
NHS Foundation Trust

Imperial College Healthcare NHS
NHS Trust

<u>HIV patient interview topic guide</u>

Overview description (recap and summary of information in the patient information sheet)

During this project we wish to find out people's own experiences of their life with HIV starting from the beginning, before you were diagnosed and going all the way up to the present. There are some specific areas we wish to cover, however you are free to discuss what you feel are important issues or areas that have been relevant in your HIV journey.

Questions:

[May or may not need to ask all the questions specifically, this is just a guide, some questions overlap]

1. Personal information – work/relationships/home situation

Can you tell me a bit about yourself….

a. What are your home circumstances?
b. Are you in a relationship/s?
c. Do you have dependents children? Parents? partner ?
d. Do you work? What do you do? Benefits?

2. Testing decisions

a. Can you tell me when you were diagnosed?
b. How did you come to be tested?
c. Where did you go for your test? Why?
d. Had you had HIV tests before, if so why?
e. What was your health like before your diagnosis?
f. Had/did you know anybody living with HIV at the time?

3. Experience of initial care

a. What happened when you were given your positive result?
b. Can you remember what information you were given at the time? Was this given in the way you wanted? Please can you tell me in detail.
c. What questions, if any, did you have for the clinical staff? Did you ask them?

For peer review only - http://bmjopen.bmj.com/site/about/guidelines.xhtml

d. Did you tell anyone / talk to anyone after you found out your results? Can you tell me a bit more about this – who were they, was it helpful to talk to them, etc.?
e. Did you access information about HIV? How? where? How has this helped?
f. What did you do after receiving your diagnosis?
g. Which HIV clinic did you go to? Why? Are you still at that clinic? Why?

4. Treatment decisions and research

a. Are you on treatment ?
b. How was the decision to start taken? Were you involved in that decision?
c. What preparation did you make?

d. (If not on treatment) What are your thoughts about going on treatment?

(If on treatment) What do you think / have you any thoughts about being on treatment?

e. (If on treatment) How do you find taking your medication?

f. Have you discussed your thoughts about treatment with anyone?
g. Do you know what recommendations there are for when people might start treatment?

h. Are you aware of the research into ARVs and treatment guidelines in the UK?
i. What do you know about them? What do you think of them?
j. How have they influenced your understanding of treatment?
k. Have they influenced your decision to start/change/stop treatment?

l. Do you take part in clinical trials? Why?

5. Clinical care

a. How often do you go to the clinic? Has that changed over the time you have been in the clinic? How?
b. Who do you see? And for what? Doctor? Consultant? Nurse? Peer support? Health Advisor? etc
c. What kind of care have you received at the clinic? Any good/bad experiences?

d. What do you think the role of the clinic is in your care?
e. What other clinical services do you access? Where? How is your care coordinated?
f. What do you think about the way in which HIV services are currently organised? Does it work for you?
g. Have they changed? In what way?
h. Are you aware of possible future changes in the services?

For peer review only - http://bmjopen.bmj.com/site/about/guidelines.xhtml

6. Primary care /Community and Social care

a. Where do you go for your primary care needs? Why? What is your experience of primary care?
b. How is your care coordinated between primary care and the clinic?
c. Do you access any social care or voluntary services? If so what? Why?
d. Do they meet your needs? If not why?
e. Have you seen any changes in what is available? If so what?

7. Living with HIV

a. Has having HIV affected your life? What are the most significant ways HIV has affected your life, and has that changed over time?
b. Do you know others living with HIV? What impact does that have on your life?
c. What kind of support have you had/needed since you were diagnosed positive? Has this changed over time?

8. Is there anything else you would like to say?

a. Given your experience of living with HIV, what would you say to others who have been recently diagnosed about what to expect?

For peer review only

For peer review only - http://bmjopen.bmj.com/site/about/guidelines.xhtml

COREQ (COnsolidated criteria for REporting Qualitative research) Checklist

A checklist of items that should be included in reports of qualitative research. You must report the page number in your manuscript where you consider each of the items listed in this checklist. If you have not included this information, either revise your manuscript accordingly before submitting or note N/A.

Topic	Item No.	Guide Questions/Description	Reported on Page No.
**Domain 1: Research team and reflexivity**			
Personal characteristics			
Interviewer/facilitator	1	Which author/s conducted the interview or focus group?	4
Credentials	2	What were the researcher's credentials? E.g. PhD, MD	1
Occupation	3	What was their occupation at the time of the study?	1
Gender	4	Was the researcher male or female?	1
Experience and training	5	What experience or training did the researcher have?	1
Relationship with participants			
Relationship established	6	Was a relationship established prior to study commencement?	4
Participant knowledge of the interviewer	7	What did the participants know about the researcher? e.g. personal goals, reasons for doing the research	4
Interviewer characteristics	8	What characteristics were reported about the inter viewer/facilitator? e.g. Bias, assumptions, reasons and interests in the research topic	1
**Domain 2: Study design**			
Theoretical framework			
Methodological orientation and Theory	9	What methodological orientation was stated to underpin the study? e.g. grounded theory, discourse analysis, ethnography, phenomenology, content analysis	5
Participant selection			
Sampling	10	How were participants selected? e.g. purposive, convenience, consecutive, snowball	4
Method of approach	11	How were participants approached? e.g. face-to-face, telephone, mail, email	4
Sample size	12	How many participants were in the study?	5
Non-participation	13	How many people refused to participate or dropped out? Reasons?	NA
Setting			
Setting of data collection	14	Where was the data collected? e.g. home, clinic, workplace	4
Presence of non-participants	15	Was anyone else present besides the participants and researchers?	4
Description of sample	16	What are the important characteristics of the sample? e.g. demographic data, date	5/6
Data collection			
Interview guide	17	Were questions, prompts, guides provided by the authors? Was it pilot tested?	5
Repeat interviews	18	Were repeat inter views carried out? If yes, how many?	NA
Audio/visual recording	19	Did the research use audio or visual recording to collect the data?	4
Field notes	20	Were field notes made during and/or after the inter view or focus group?	5
Duration	21	What was the duration of the inter views or focus group?	4
Data saturation	22	Was data saturation discussed?	N/A
Transcripts returned	23	Were transcripts returned to participants for comment and/or	NA

For peer review only

Topic	Item No.	Guide Questions/Description	Reported on Page No.
		correction?	
**Domain 3: analysis and findings**			
Data analysis			
Number of data coders	24	How many data coders coded the data?	5
Description of the coding tree	25	Did authors provide a description of the coding tree?	NA
Derivation of themes	26	Were themes identified in advance or derived from the data?	5
Software	27	What software, if applicable, was used to manage the data?	5
Participant checking	28	Did participants provide feedback on the findings?	NA
Reporting			
Quotations presented	29	Were participant quotations presented to illustrate the themes/findings? Was each quotation identified? e.g. participant number	6,7,8,9
Data and findings consistent	30	Was there consistency between the data presented and the findings?	9,10,11
Clarity of major themes	31	Were major themes clearly presented in the findings?	9,10,11
Clarity of minor themes	32	Is there a description of diverse cases or discussion of minor themes?	9,10,11

Developed from: Tong A, Sainsbury P, Craig J. Consolidated criteria for reporting qualitative research (COREQ): a 32-item checklist for interviews and focus groups. *International Journal for Quality in Health Care*. 2007. Volume 19, Number 6: pp. 349 – 357

Once you have completed this checklist, please save a copy and upload it as part of your submission. DO NOT include this checklist as part of the main manuscript document. It must be uploaded as a separate file.

For peer review only

BMJ Open

Patient perspectives on the HIV continuum of care in London: a qualitative study of people diagnosed between 1986 and 2014

Journal:	*BMJ Open*
Manuscript ID	bmjopen-2017-020208.R2
Article Type:	Research
Date Submitted by the Author:	14-Feb-2018
Complete List of Authors:	Bruton, Jane; Imperial College Lodon, Department of Infectious Disease Epidemiology<br>Rai, Tanvi; Imperial College Lodon, Department of Infectious Disease Epidemiology<br>Day, Sophie; Imperial College Lodon, Department of Infectious Disease Epidemiology; Goldsmiths University of London, Anthropology<br>Ward, Helen; Imperial College London, Department of Infectious Disease Epidemiology
**Primary Subject Heading**:	HIV/AIDS
Secondary Subject Heading:	Qualitative research, Health services research, Patient-centred medicine
Keywords:	HIV & AIDS < INFECTIOUS DISEASES, QUALITATIVE RESEARCH, PATIENT EXPERIENCE, HIV CARE CONTINUUM

SCHOLARONE™
Manuscripts

Patient perspectives on the HIV continuum of care in London: a qualitative study of people diagnosed between 1986 and 2014

Jane Bruton RN, BA, MA Medical Anthropology; Tanvi Rai PhD Public Health, MPH, BA, BSc; Sophie Day, PhD, BA, MA Anthropology; BSc Helen Ward PhD, FRCP, FFPH, MBChB, MSc Epidemiology.

Corresponding author:
Pamela Jane Bruton,
Department of Infectious Disease Epidemiology,
School of Public Health,
Imperial College,
St Mary's Campus,
Norfolk Place,
London.
W2 1PG
jbruton@ic.ac.uk
0207 5943464

Co-authors:
Rai, Tanvi: Imperial College London, Department of Infectious Disease Epidemiology.
Day, Sophie: Imperial College London, Department of Infectious Disease Epidemiology and Goldsmiths College
Ward, Helen: Imperial College London, Department of Infectious Disease Epidemiology.

Word count: Main text 4051

For peer review only

Abstract

Objectives: To describe the experiences of the HIV treatment cascade of diagnosis, engagement with care and initiation of treatment, from the perspective of patients; we explored whether this differed according to the year of their diagnosis, for example whether they had experienced HIV care in the pre-treatment era.

Design: Qualitative interview study with framework analysis

Setting: Two large HIV adult outpatient clinics in central London.

Participants: 52 HIV positive individuals, 41 men, 11 women, purposively sampled to include people who had been diagnosed at different stages in the history of the epidemic classified as four 'generations': pre-1996 (pre-ART), 1997 to 2005 (complex ARTs), 2006 to 2012 (simpler ARTs), 2013 onwards (recent diagnoses).

Results: Some important differences were identified; for earlier generations, the visible illness and deaths from AIDS made it harder to engage with care following diagnosis. Subsequent decisions about starting treatment were deeply influenced by the fear of severe side-effects from early antiretroviral therapy (ART). However, despite improvements in ART and life expectancy over the epidemic we found a striking similarity across participants' accounts of the key stages of the care continuum, regardless of when they were diagnosed. Diagnosis was a major traumatic life event for almost everyone, fear of testing positive or having low self-perceived risk affected the timing of testing and diagnosis, engaging with care was facilitated by a flexible approach from services/clinicians and initiating treatment was a major life decision.

Conclusion: We found patients' experiences are influenced by when they were diagnosed, with earliest cohorts facing substantial challenges. However, being diagnosed with HIV and starting treatment continue to be significant life-altering events even in the era of effective, simple treatments. Despite the advances of biomedical treatment services should continue to recognise the needs of patients for whom the diagnosis and treatment remain significant challenges.

For peer review only

For peer review only - http://bmjopen.bmj.com/site/about/guidelines.xhtml

Strengths

- The large number of participants, at two different clinics and the inclusion of a range of people with HIV, broadly similar to the clinic cohorts, across four HIV 'generations'.
- The interview format allowed us to explore factors important to participants rather than asking about pre-defined concerns.

Limitations

- An imbalance between generations, with larger numbers of women in the earliest two generations and only one woman in the generation recently diagnosed. This means that some of our conclusions about generations may also reflect gendered differences.
- Our recruitment methods meant we were not able to explore the experiences of those who remain outside care.
- Limitation of the single interview format means people were having to recall past experiences, some of which were three decades ago. This may have introduced recall bias with subsequent experience colouring earlier reports.

For peer review only

For peer review only - http://bmjopen.bmj.com/site/about/guidelines.xhtml

INTRODUCTION

The Human Immunodeficiency Virus (HIV) continuum of care provides stepwise estimates for the stages of engagement in care for people living with HIV (PLHIV).(1) The Joint United Nations Programme on HIV and AIDS (UNAIDS) goal to end the Acquired Immune Deficiency Syndrome (AIDS) epidemic by 2020 stipulates a target of 90% of all people with HIV be diagnosed, of whom 90% are on antiretroviral therapy (ART), of whom 90% (73% of PLHIV) are virally suppressed.(2) The United Kingdom (UK) has one of the best outcomes for HIV treatment and care in the world with an estimated 78% of PLHIV having an undetectable viral load.(3, 4) However the statistics do not and cannot tell us about the patient experience of passing through these stages of care whether it be good or bad. The continuum essentially measures the success of programmes from a provider rather than a patient perspective.(5)

Flowers argues that there is a tension between the certainty and confidence of a linear HIV pathway, associated with ideas of clinical efficacy, and patient experiences of diagnosis and prognosis, which can be full of uncertainty.(4) With the evolution of modern ARTs we have witnessed the transformation of HIV from an acute life-threatening infection to a treatable chronic condition and a concurrent evolving of the care continuum. However, it is unclear whether this change is reflected in patients' own experiences of passing through each of the stages of care. For example, has the moment of diagnosis become any less traumatic, and have decisions about starting treatment become simpler for patients? Analysis of patient narratives, historically and currently, may help to highlight significant factors for patients in the care continuum. This is particularly important in a climate of National Health Service (NHS) restructuring in the UK, which has led to reductions in non-clinical services and streamlining of care. To this end we explored the patient perspectives on the care continuum. And we hypothesized that patients' experiences of, and engagement with, care would differ according to what point in the epidemic they were diagnosed.

METHODS

We undertook a qualitative study of people attending two public HIV clinics in London that have provided care since the start of the epidemic; they were also chosen for their large size and diversity, in terms of demographics. They are both specialist HIV clinics linked to sexual health (genitourinary medicine) services. Care is provided by physician-led multidisciplinary teams where patients have a named consultant. HIV care in the UK is free and open access, allowing patients to register at their clinic of choice. We used a purposive sampling method to recruit patients with a range of experiences. To reflect the evolution of ART, we identified four 'generations' according to time of diagnosis: pre-1996 (pre-ART), 1997 to 2005 (complex ARTs), 2006 to 2012 (simpler ARTs), 2013

For peer review only - http://bmjopen.bmj.com/site/about/guidelines.xhtml

onwards (recent diagnoses). Within each generation, we aimed to include people with a range of characteristics, including, gender, exposure, age and ethnicity. Participants were recruited opportunistically by researchers attending clinical services, and through fliers and digital advertising in clinical areas. Recruitment was periodically checked against the recruitment matrix and under-represented groups/strata targeted.

Patients were provided with information and gave written consent. Interviews took place in private rooms in or near the clinics or at the patient's home; they were recorded and transcribed, and lasted between 60 and 90 minutes. The interviews were semi-structured and carried out by one male (CH) and three female researchers (JB, TR, JR), three of whom had clinical backgrounds. The interviews were based on a topic guide (see supplementary file) informed by a focus group of PLHIV who assisted in designing the research. We invited participants to recall their initial diagnosis and describe key points in their HIV journey including testing, disclosure, support, engaging with care, starting treatment, medication adherence, work and social life. Field notes were written after the interviews.

Transcripts were uploaded to NVivo, a qualitative data analysis software package. Using Framework analysis, we developed key themes through a systematic process which involved reading, rereading, coding and summarising the transcripts; and subsequent in-depth analysis of the dataset.(6)Final themes were discussed in the research group (HW, JB, SD, TR) and further analysed in relation to the existing literature.

Ethical approval was obtained from the National Research Ethics Service (NRES) (reference number 14/WM/0147) in May 2014, and research governance approval obtained from the local sites.

RESULTS

Fifty-two patients were recruited, 25 at one clinic and 27 at the other. The sample included 41 men and 11 women; 37 men acquired HIV through sex with other men (MSM), the rest through heterosexual contact (n=14) or injection drug use (n=1). There were 11 in generation 1, 14 generation 2, 17 generation 3 and 10 generation 4. The characteristics of the study participants alongside those of the clinic population for 2014 are shown in Table 1.

Table 1. Study sample characteristics compared with the clinic cohorts

	Clinic A				Clinic B			
	Study sample		2014 Cohort		Study sample		2014 Cohort	
**Gender**								
Male	18	72.0%	2459	77.5%	23	85.2%	7743	90.3%
Female	7	28.0%	715	22.5%	4	14.8%	830	9.7%

For peer review only - http://bmjopen.bmj.com/site/about/guidelines.xhtml

**Age**								
18-24	0	0.0%	124	3.9%	0	0.0%	186	2.2%
25-34	4	16.0%	465	14.7%	3	11.1%	1729	20.2%
35-49	16	64.0%	1502	47.3%	14	51.9%	4164	48.6%
50+	5	20.0%	1083	34.1%	10	37.0%	2494	29.1%
**Ethnicity**								
White	14	56.0%	1541	48.6%	22	81.5%	6401	74.7%
Black African	5	20.0%	720	22.7%	5	18.5%	778	9.1%
Black Caribbean	0	0.0%	114	3.6%	0	0.0%	201	2.3%
Other/Mixed	6	24.0%	746	23.5%	0	0.0%	1132	13.2%
Not reported	0	0.0%	53	1.7%	0	0.0%	61	0.7%
**Exposure route**								
Sex between men	15	60.0%	1971	62.1%	22	81.5%	6776	79.0%
Heterosexual contact	9	36.0%	1037	32.7%	5	18.5%	1209	14.1%
Injecting drug use	1	4.0%	52	1.6%	0	0.0%	109	1.3%
Other	0	0.0%	112	3.5%	0	0.0%	46	0.5%
Undetermined	0	0.0%	2	0.1%	0	0.0%	433	5.1%
**Year of diagnosis**								
Pre-1997	6	24.0%	638	20.1%	5	18.5%	1399	16.3%
1997-2005	6	24.0%	1234	38.9%	8	29.6%	2580	30.1%
2006-2012	7	28.0%	986	31.1%	10	37.0%	3199	37.3%
2013 onwards	6	24.0%	316	10.0%	4	14.8%	1395	16.3%
Total			3174				8573	

The generation samples differed somewhat by gender and acquisition: the women were concentrated in generations 1 and 2 (n=6 and 4, respectively), and MSM in generations 3 and 4 (n=16 and 8, respectively).

"Becoming positive" – the impact of HIV diagnosis

The experience of receiving an HIV diagnosis was similar across the generations. First reactions were generally of shock and fear of death, irrespective of generation. Alan (pseudonym), diagnosed in 1991, knew nothing about HIV and had not tested before. He remembers vividly the time he received his result:

> *"I could hear myself saying 'I'm going to die'. Not verbally but in my mind, 'I'm going to die, I'm going to die'"* (gen1, MSM)

For peer review only - http://bmjopen.bmj.com/site/about/guidelines.xhtml

Sylvia, diagnosed 2001, was “totally devastated”:

> *“… I didn’t see myself going back and doing my Master’s degree for what reason am I going back to do that if I have maybe five years to live”* (gen 2, woman)

Roger, diagnosed more than 20 years later, reported several previous tests and considered himself well-informed. However, his principal concern on receiving a positive diagnosis was also about life expectancy:

> *“But even I was not certain. Certainty is the wrong word. I was under the illusion that my expiry date was stamped on me now”* (gen4, MSM)

Fear of a positive result was a key factor in delayed diagnosis for several MSM in all generations, who reported concerns about the impact of HIV on their lives. They were aware of their risk, and described feeling relieved at diagnosis as HIV had been *“hanging over them”* for years; the diagnosis confirmed their suspicions. William, who had never tested before, presented with symptoms:

> *“[I had been] burying my head in the sand. I guess I knew I had it but didn’t, at the same time, want it confirmed”* (gen2, MSM)

Brian (Gen4, MSM), recently diagnosed, had “*spent on and off probably eight years thinking about it*” He felt he had “*done all the thinking before*” … so although disappointed, he was also relieved.

Most other participants, particularly heterosexual men and women, were not expecting a positive result and had not requested an HIV test. They were diagnosed either following ongoing symptoms of ill health or having presented for a general sexual health check-up.

None of the women had been diagnosed during pregnancy; most were diagnosed before ante-natal screening became routine in the UK (1999). For example, Olivia, diagnosed in 1998, had not been tested in pregnancy. Her six-month old baby became sick, and both baby and husband were then diagnosed with HIV but she did not believe she had HIV and delayed testing for several weeks

> *“Me I don’t have HIV because I never went with other men”* (gen2, woman)

The response and level of support offered by clinicians at this critical time were important to participants’ immediate wellbeing and influenced what happened next, including continuing engagement in care. Martha remarked:

> *“I remember how lovely [name of clinician] was and I’ve always said I could never wish for a better person to ever tell me or try to guide me, or to reassure me more than what she did because she was perfect”* (Gen4, woman)

While most experiences were positive, there were some exceptions. Paul (gen3, MSM) had regularly tested negative but continued to take risks. Testing positive in 2010 was totally unexpected, leaving

For peer review only - http://bmjopen.bmj.com/site/about/guidelines.xhtml

him "*numb with shock*", and he did not feel that he was supported appropriately. The clinician who gave him the diagnosis seemed "[to be on] *autopilot because he had seen people like me before*" and was "*working to his own agenda*". Despite Paul's obvious distress, the clinician asked him to ring potential contacts during the consultation. Further, when the clinician said: "*Oh this can be managed, don't worry*", Paul interpreted this to mean 'managed to his death'. After two weeks of acute anxiety, Paul contacted a friend who was able to reassure him about treatment and the care pathway. Similar experiences led other participants to feel vulnerable, isolated and slow to accept their diagnosis.

"Becoming an HIV patient": Developing a relationship with clinic and clinician

Once diagnosed, participants described a sense of reassurance about being in the "best hands*"*, managed by experts in HIV medicine. The majority across the generations described strong relationships with their clinicians and valued seeing the same person each visit. It felt "*like a partnership*" with "*someone you can tell anything*", who knew them and their entire history, ensuring that care went beyond just the clinical management of HIV: "*We seriously talk about how I am not just what my CD4 count is*".

However, some had not developed a trusting relationship. Marty (gen3, MSM), for example, diagnosed HIV in 2012, was not eligible for treatment under guidelines at that point. He described anxiety about this lack of treatment, feeling that it adversely affected pre-existing mental health problems which were not addressed by his clinicians. He attended two different clinics and was on the verge of dropping out of care when, as he recounted: *"I basically rescued myself*". His friend recommended a clinician: "*She got me just like that thank god, thank god…I finally found and she was willing to fight my case*".

Another participant, Peter, diagnosed 2009, reported changing HIV clinics within three months of diagnosis. He recalled a series of mistakes, miscommunication and a "dehumanising" clinic environment. Losing trust in clinicians and the service, he finally gained confidence from attending a support group and moved his care:

> "*I remember that I said, it was like falling off a building… I'm slowly falling backwards as the virus increases. It felt like they were holding a blanket at the bottom to catch me but it felt like they were holding it in the wrong place. I was being asked to trust"* (gen3, MSM)

For peer review only - http://bmjopen.bmj.com/site/about/guidelines.xhtml

All participants valued continuity of care although two recently diagnosed felt that it was not always necessary to see the same clinician. But, continuity was affected by what some described as the very busy clinics limiting the time for consultations and impeding communication.

All participants were in care at the time of the interview but some described having stayed away in the past. Two of six women diagnosed before 1996 had dropped out of care for several years. Given the lack of effective treatment, they had found clinic visits depressing and preferred to keep away until they became sick. Marie (gen1, woman) explained, *"I didn't want a life where I just would go to tests and I am scared and they had nothing to offer".* Alison (gen1, woman) described the *"terrible situation"* at the clinic, where she saw young gay men, couples, where one would be fit and the other *"in a wheelchair, a skeleton".* She felt sorry for the doctors - *"there were all these poor young doctors with nothing to offer and seeing these very ill people."*

Seven of all those diagnosed since ARTs became available described occasional or multiple lapses in attendance; these were generally explained by issues external to the clinic such as recreational drug use, household disruption, mental health problems or competing co-morbidities. Re-engagement with care was easier when their clinician actively reached out; for example, some consultants had telephoned patients when they missed appointments and one woman (gen1) described interventions of this kind as being "*like my family*". Even though not all patients were contacted when they did not attend services, and one expressed surprise that no-one had tried, all found their way back into care.

"Becoming medicalised": Starting treatment

Almost all (48) of the participants were on treatment; one had stopped medication because of drug interactions and three chose to remain off treatment. Despite the simplification of regimens, participants in all four generations found the decision to start treatment a significant life event.

For earlier generations, the decision had been complex because of toxicity related to ARTs. Some refused treatment contrary to medical advice. Alison, described earlier, recalled, "*In the waiting rooms people said, 'don't take it, it will kill you', so I refused that (AZT*)". Others felt well without treatment like Marie who was diagnosed in 1986 but only started ARTs 25 years after diagnosis when her CD4 count crashed. She felt she had no choice, "I fought it all this time on my own, and then finally I had to give in and take a pill. That was kind of depressing".

For peer review only - http://bmjopen.bmj.com/site/about/guidelines.xhtml

Those diagnosed more recently found it easier to decide to begin treatment, but it was still a significant moment. Tim, diagnosed in 2012, was aware of the latest research and asked to start treatment immediately, even for him, the 'treatment appointment' was a sobering experience:

> *"[It was] the only time there was a tear. I just thought, God this is a new chapter now. This is a new chapter in my life. I am going to have to take this pill for the rest of my life".* (, gen 3 MSM)

Brian, diagnosed in 2013, sums up some of the issues that participants said they had considered when deciding to start medication:

> *"Well the impact it would have on my life, the damage it would do to my body. Would I cope with the medication? Would I be able to continue working? Because so many people have side effects initially and it takes them a long time to get over. I had a lot of responsibility at work and I couldn't actually manage responsibility well enough once on medication. Would I be able to take the medication on time? Would life's pressures allow me to do what I needed to do? And so on"* (gen 4, MSM)

The three participants not on treatment described feeling healthy and wished to remain drug free for as long as possible.

For many participants, HIV occupied only a small part of their lives but daily medications proved to be a constant reminder of their status even if only for a moment each day. Martin, diagnosed in 2014, described his relationship with his medication:

> *"It's strange sometimes because you look at this pill and you think between you and this little pill lies – it's keeping you alive. And I have never had a pill to take like that before. So, it's very strange. It's my friend and foe at the same time"* (gen 4, MSM)

DISCUSSION

We have found that patients' experiences of, and engagement with, care are influenced by the point at which they were diagnosed, with the earliest cohorts facing substantial challenges. However, we have also found that being diagnosed with HIV and starting treatment continue to be significant life-altering events even in the era of effective and simple treatments. This study brings new insights which are important when considering how future services should be provided.

The revolution in HIV treatment over three decades means it can now be described as a chronic, manageable condition.(7, 8) In our study, all our patients were virally supressed having passed

For peer review only - http://bmjopen.bmj.com/site/about/guidelines.xhtml

through all the stages of the continuum and arrived on the other side. However, patients' recall of their experience of navigating this journey revealed a range of quite complex issues faced by them at different points, reminding us that many challenges remain in the successful provision of HIV care. We hypothesized that the revolution in treatment would have an impact on the experience of the different diagnostic generations moving through the care continuum, with those diagnosed more recently having a smoother journey. We identified some important differences; the visible illness and deaths from AIDS made it harder for earlier generations to engage with or remain in care and some dropped out, returning only when ill. Decisions about treatment were particularly difficult for the early generations, for whom the association of treatment with severe side effects remained strong until more recently and who expressed pride in, and determination to, remaining well without treatment.

However, our primary finding was a striking similarity across participants' accounts of key stages of the HIV care continuum: diagnosis was a major, traumatic life event for almost everyone, and anticipation of an HIV-positive status affected the timing of testing and diagnosis. Engagement with care was facilitated by a responsive, flexible approach on the part of services and clinicians, starting with the way the positive diagnosis was handled. Finally, initiating treatment was a major life decision even when recommended by protocol and considered straightforward by clinicians.

Despite the drive to normalise HIV testing through simplified sampling, reduced pre-test discussion and expanded test settings,(9, 10) (11) receiving an HIV diagnosis remained a significant shock for most participants irrespective of generation, sexuality or gender, as suggested elsewhere.(12-15) Moreover, we found that none of the heterosexual participants were expecting a positive result, a finding which also applied to some of the MSM participants. Resonating with other studies.(13, 16, 17) , we found that fear of imminent death and experience of profound distress did not change, despite the availability of effective and less toxic treatments. This fear, often coupled with a fear of social exclusion and rejection, led some MSM participants who suspected they were positive to delay testing.(18) Bury (1982) usefully describes this experience of illness and especially chronic illness as "biographical disruption". When everyday life and its meanings are turned upside down, relationships and social networks are disrupted and plans for the future have to be re-examined. (19) Participants described this type of disruption at diagnosis, whether HIV was considered an acute infection or a chronic condition.

A clinician's approach to a patient with a positive result is considered critical to patients' experiences and may be more important than other aspects of the testing process.(12, 15, 17, 20, 21) We found that negative experiences at this critical point affected immediate well-being and further contact

For peer review only - http://bmjopen.bmj.com/site/about/guidelines.xhtml

with services. The impact of those initial encounters, both good and bad, left participants with lasting impressions throughout their journey, thus demonstrating the importance of establishing trust between clinician and patient as a firm foundation for good retention in care.(22-24) Women, a minority in the clinics and, in our sample mainly from the earlier generations, faced particular challenges in engaging in care. This made the establishment of a trusting relationship with their clinician all the more important to managing their quality of life with HIV. Moreover, the importance of that relationship for participants was underlined by their willingness to change their treatment centre until they found what they perceived as a good clinician/patient relationship. The UK policy of open access to any clinic through self-referral may be another explanation for high levels of retention in care.

The prospect of treatment for life, to sustain life, was a major life event. Currently the moves towards starting treatment at diagnosis, the test-and-treat model, is based on the confidence of biomedicine in HIV management, but may be at odds with patient concerns.(25) British HIV Association (BHIVA) interim guidelines 2016 recommend starting treatment on all diagnosed with HIV regardless of CD4 count and continue to recognise that social, psychological, cultural, and economic factors can adversely affect adherence and treatment outcomes.(26) Starting medication on the same day as, or soon after, diagnosis when individuals may be distressed by the positive result could preclude a meaningful discussion of the patient's 'readiness to start'. Persson's 2016 study of patients not on ARTs found similar barriers and concerns: for example, logistics of starting life-long medication, fear of long term side effects and desire to stay drug-free whilst healthy.(27)

Considering ARTs was another point at which some participants anticipated 'biographical disruption' which deterred them from starting treatment.

The study's strengths are in the large number of participants at two different clinics, the inclusion of a range of people with HIV broadly similar to the cohorts seen at these clinics, and diagnosed across the four generations. The semi-structured interview format allowed us to explore factors important to participants rather than asking about pre-defined concerns. However, our study sample was imbalanced between generations, with larger numbers of women in the earliest two generation and only one of those more recently diagnosed. This means that some of our conclusions about generation may also reflect gendered differences. Our recruitment methods that relied on recruitment from the two clinics meant we were not able to explore the experiences of those who remain outside care. The study is also limited by the single interview format which meant people were having to recall past experiences, some of which were three decades ago. This may have introduced recall bias with subsequent experience colouring earlier reports; moreover, earlier

For peer review only - http://bmjopen.bmj.com/site/about/guidelines.xhtml

generations had more time to have disengaged and re-engaged with care. The focus of the study on a particular model of care in London limits the generalisability of our findings to other settings.

The evolution of simpler treatments has been accompanied by a reconfiguration of the care pathway. In London, HIV clinics are increasingly narrowly focused on HIV and HIV-specific medications and clinicians are not authorised to provide more holistic medical care.(28) In practice, this has led to less frequent clinic visits, a shift to virtual “e-clinics”, and greater links with, and reliance on, general practitioners. The impact of this on the care continuum is unclear. Continuity of clinician, the atmosphere in the clinic and good communication are recognised to be key issues for patients. (29, 30)

There is increasing recognition that viral suppression is not the final goal for people who are now living longer with HIV.(31-33) Lazarus et al (2016) have called for a ‘fourth 90’ “providing an explicit target for health- related quality of life”(31). In December2017 these concerns were embodied in policy recommendations from the European Parliament calling for an integrated and patient-centred approach to long term HIV care ensuring that services are meeting this challenge. (34) Our study illustrates that the patient journey is complex and personalised care should not be lost with streamlining pathways. All our participants were virally suppressed but that can hide the reality of their reliance on the caregivers to help maintain that stability.

A personal, holistic approach has been a hallmark of HIV care since the beginning of the epidemic. It is important that major advances in biomedical treatment do not undermine the care continuum through a loss of care that meets the complex needs of patients, for whom HIV diagnosis and treatment remain significant challenges requiring supportive and flexible care.

For peer review only

For peer review only - http://bmjopen.bmj.com/site/about/guidelines.xhtml

Acknowledgements: Thanks to participants for sharing their time and stories, to Jane Rowlands and Chris Higgs for conducting some of the interviews and the staff of both clinics for facilitating the study.

Contributership statement: All four authors, J. Bruton, T. Rai, S. Day and H. Ward, were involved in the design of the study. J. Bruton and T. Rai completed the initial analysis and interpretation of the data and subsequent discussion and analysis was undertaken by all 4 authors through team discussion. J. Bruton completed the first draft of the manuscript. All four authors participated in critiquing and revising the draft manuscript and all approved the final draft.
All four authors accept accountability for the submitted paper.
T. Rai and J. Bruton only undertook the interviews alongside collaborators J. Rowlands and C.Higgs.

Funders: Funded by grants from Imperial NIHR Biomedical Research Centre, P35771, the Imperial College Healthcare Charity, P44223, and supported by the St Stephens AIDS Trust. No pharmaceutical grants were received in the development of this study.

Competing Interests: There are no competing interests

Data sharing statement: The interview transcripts are anonymised and stored securely at Imperial College London on a password protected database on a password protected computer. The participants were assured of the security of their data and they would remain confidential.

References

1. Gardner EM, McLees MP, Steiner JF, del Rio C, Burman WJ. The Spectrum of Engagement in HIV Care and its Relevance to Test-and-Treat Strategies for Prevention of HIV Infection. Clinical Infectious Diseases. 2011;52(6):793-800.
2. UNAIDS. UNAIDS. 90-90-90 - an ambitious treatment target to help end the AIDS epidemic (accessed June 21, 2017) http://www.unaids.org/en/resources/documents/2014/90-90-90. 2014.
3. Kirwan PD CC, Brown AE., Gill ON.,Delpech VC. and contributors. HIV in the UK: 2016 Report. London: Public Health England; 2016.
4. Flowers P, Davis MM, Larkin M, Church S, Marriott C. Understanding the impact of HIV diagnosis amongst gay men in Scotland: an interpretative phenomenological analysis. Psychology & health. 2011;26(10):1378-91.
5. Christopoulos KA, Massey AD, Lopez AM, Geng EH, Johnson MO, Pilcher CD, et al. "Taking a half day at a time:" patient perspectives and the HIV engagement in care continuum. AIDS patient care and STDs. 2013;27(4):223-30.
6. Gale NK, Heath G, Cameron E, Rashid S, Redwood S. Using the framework method for the analysis of qualitative data in multi-disciplinary health research. BMC Medical Research Methodology. 2013;13(1):117.

For peer review only - http://bmjopen.bmj.com/site/about/guidelines.xhtml

7. Deeks SG, Lewin SR, Havlir DV. The End of AIDS: HIV Infection as a Chronic Disease. Lancet. 2013;382(9903):1525-33.
8. Yehia BR, Gebo KA, Hicks PB, Korthuis PT, Moore RD, Ridore M, et al. Structures of Care in the Clinics of the HIV Research Network. AIDS patient care and STDs. 2008;22(12):1007-13.
9. Ogaz D. FM, Connor N., Gill ON. and contributors. HIV testing in England:2016 report. London England: Public Health England; 2016.
10. Rayment M, Thornton A, Mandalia S, Elam G, Atkins M, Jones R, et al. HIV Testing in Non-Traditional Settings – The HINTS Study: A Multi-Centre Observational Study of Feasibility and Acceptability. PLOS ONE. 2012;7(6):e39530.
11. Wise J. Guidelines call for HIV testing to be "normalised". BMJ. 2008;337.
12. Roth NL, Nelson MS. HIV diagnosis rituals and identity narratives. AIDS Care. 1997;9(2):161-80.
13. Stevens PE, Hildebrandt E. Life changing words: women's responses to being diagnosed with HIV infection. ANS Advances in nursing science. 2006;29(3):207-21.
14. Stevens PE, Tighe Doerr B. Trauma of discovery: women's narratives of being informed they are HIV-infected. AIDS Care. 1997;9(5):523-38.
15. Anderson M, Elam G, Gerver S, Solarin I, Fenton K, Easterbrook P. "It took a piece of me": initial responses to a positive HIV diagnosis by Caribbean people in the UK. AIDS Care. 2010;22(12):1493-8.
16. Meursing K, Sibindi F. HIV counselling — a luxury or necessity? Health Policy and Planning. 2000;15(1):17-23.
17. Christopoulos KA, Massey AD, Lopez AM, Hare CB, Johnson MO, Pilcher CD, et al. Patient Perspectives on the Experience of Being Newly Diagnosed with HIV in the Emergency Department/Urgent Care Clinic of a Public Hospital. PLOS ONE. 2013;8(8):e74199.
18. Clifton S, Nardone A, Field N, Mercer CH, Tanton C, Macdowall W, et al. HIV testing, risk perception, and behaviour in the British population. AIDS (London, England). 2016;30(6):943-52.
19. Bury M. Chronic illness as biographical disruption. Sociology of health & illness. 1982;4(2):167-82.
20. Worthington C, Myers T. Desired elements of HIV testing services: test recipient perspectives. AIDS patient care and STDs. 2002;16(11):537-48.
21. Hult JR, Maurer SA, Moskowitz JT. "I'm sorry, you're positive": a qualitative study of individual experiences of testing positive for HIV. AIDS Care. 2009;21(2):185-8.
22. Kempf MC, McLeod J, Boehme AK, Walcott MW, Wright L, Seal P, et al. A qualitative study of the barriers and facilitators to retention-in-care among HIV-positive women in the rural southeastern United States: implications for targeted interventions. AIDS patient care and STDs. 2010;24(8):515-20.
23. Yehia BR, Stewart L, Momplaisir F, Mody A, Holtzman CW, Jacobs LM, et al. Barriers and facilitators to patient retention in HIV care. BMC Infectious Diseases. 2015;15(1):246.
24. Engelhard EA, Smit C, Van Sighem A, Reiss P, Nieuwkerk PT, Kroon FP, et al. Impact of HIV care facility characteristics on the cascade of care in HIV-infected patients in the Netherlands. AIDS (London, England). 2016;30(2):301-10.
25. Newman CE. Appreciating doubts about HIV medicine2015.
26. BHIVA. British HIV Association guidelines for the treatmnet of HIV-1- positive adults with antiretroviral therapy. 2015 (2016 interim update) 2016.

27. Persson A, Newman CE, Mao L, de Wit J. On the Margins of Pharmaceutical Citizenship: Not Taking HIV Medication in the "Treatment Revolution" Era. Medical anthropology quarterly. 2016;30(3):359-77.
28. Miller R. GJ. Commissioning London's HIV Services. Civitas (Institute for the Study of Civil Society); 2011.
29. Weatherburn P. KP, Reid D., Hammond G., Jessup K. My care I care: A study of what people with HIV value about NHS HIV Services across London. Sigma Research; 2013.

For peer review only - http://bmjopen.bmj.com/site/about/guidelines.xhtml

30. Haggerty JL, Reid RJ, Freeman GK, Starfield BH, Adair CE, McKendry R. Continuity of care: a multidisciplinary review. BMJ: British Medical Journal. 2003;327(7425):1219.
31. Lazarus JV, Safreed-Harmon K, Barton SE, Costagliola D, Dedes N, del Amo Valero J, et al. Beyond viral suppression of HIV – the new quality of life frontier. BMC Medicine. 2016;14(1):94.
32. Highlights from the BHIVA Satellite Symposium, IAS Conference, Paris, France, July 2017: 'Tougher times: adapting to increasing demand with declining resources'. Journal of Virus Eradication. 2017;3(4):250-2.
33. Martel K CJ, Auberbach J. Looking Beyond Viral Suppression: Findings from The Well Project's User Survey on Factors Influeincing the Health, Well-being, and Quality of Life of Women Living with HIV [IAS 2017 Poster]. 2017 [Available from: http://www.thewellproject.org/news-press/looking-beyond-viral-suppression-findings-well-project%E2%80%99s-2016-user-survey-factors.
34. HIV Outcomes, Beyond Viral Suppression. Recommendations Launched at the European Parliament. 2017 [cited 03/01/2018]. [cited 03/01/2018]. Available from: http://hivoutcomes.eu/wp-content/uploads/2017/11/HIV_Booklet_FINAL-DIGITAL-version.pdf.

For peer review only

For peer review only - http://bmjopen.bmj.com/site/about/guidelines.xhtml

Key statements

- A qualitative study understanding the patients' perspective of their experience of the HIV care continuum
- The diverse study cohort spans 4 'generations' diagnosed at different time points between 1986 and 2014 reflecting the evolution of ARTs
- There are striking similarities of experience and significance at key points on the continuum: diagnosis, engaging in care and starting treatment
- Despite advances of biomedical treatment clinical services should continue to recognise the needs of patients for whom HIV diagnosis and treatment remains a significant challenge

For peer review only

For peer review only - http://bmjopen.bmj.com/site/about/guidelines.xhtml

COREQ (COnsolidated criteria for REporting Qualitative research) Checklist

A checklist of items that should be included in reports of qualitative research. You must report the page number in your manuscript where you consider each of the items listed in this checklist. If you have not included this information, either revise your manuscript accordingly before submitting or note N/A.

Topic	Item No.	Guide Questions/Description	Reported on Page No.
**Domain 1: Research team and reflexivity**			
Personal characteristics			
Interviewer/facilitator	1	Which author/s conducted the interview or focus group?	4
Credentials	2	What were the researcher's credentials? E.g. PhD, MD	1
Occupation	3	What was their occupation at the time of the study?	1
Gender	4	Was the researcher male or female?	1
Experience and training	5	What experience or training did the researcher have?	1
Relationship with participants			
Relationship established	6	Was a relationship established prior to study commencement?	4
Participant knowledge of the interviewer	7	What did the participants know about the researcher? e.g. personal goals, reasons for doing the research	4
Interviewer characteristics	8	What characteristics were reported about the inter viewer/facilitator? e.g. Bias, assumptions, reasons and interests in the research topic	1
**Domain 2: Study design**			
Theoretical framework			
Methodological orientation and Theory	9	What methodological orientation was stated to underpin the study? e.g. grounded theory, discourse analysis, ethnography, phenomenology, content analysis	5
Participant selection			
Sampling	10	How were participants selected? e.g. purposive, convenience, consecutive, snowball	4
Method of approach	11	How were participants approached? e.g. face-to-face, telephone, mail, email	4
Sample size	12	How many participants were in the study?	5
Non-participation	13	How many people refused to participate or dropped out? Reasons?	NA
Setting			
Setting of data collection	14	Where was the data collected? e.g. home, clinic, workplace	4
Presence of non-participants	15	Was anyone else present besides the participants and researchers?	4
Description of sample	16	What are the important characteristics of the sample? e.g. demographic data, date	5/6
Data collection			
Interview guide	17	Were questions, prompts, guides provided by the authors? Was it pilot tested?	5
Repeat interviews	18	Were repeat inter views carried out? If yes, how many?	NA
Audio/visual recording	19	Did the research use audio or visual recording to collect the data?	4
Field notes	20	Were field notes made during and/or after the inter view or focus group?	5
Duration	21	What was the duration of the inter views or focus group?	4
Data saturation	22	Was data saturation discussed?	N/A
Transcripts returned	23	Were transcripts returned to participants for comment and/or	NA

For peer review only

Topic	Item No.	Guide Questions/Description	Reported on Page No.
		correction?	
**Domain 3: analysis and findings**			
Data analysis			
Number of data coders	24	How many data coders coded the data?	5
Description of the coding tree	25	Did authors provide a description of the coding tree?	NA
Derivation of themes	26	Were themes identified in advance or derived from the data?	5
Software	27	What software, if applicable, was used to manage the data?	5
Participant checking	28	Did participants provide feedback on the findings?	NA
Reporting			
Quotations presented	29	Were participant quotations presented to illustrate the themes/findings? Was each quotation identified? e.g. participant number	6,7,8,9
Data and findings consistent	30	Was there consistency between the data presented and the findings?	9,10,11
Clarity of major themes	31	Were major themes clearly presented in the findings?	9,10,11
Clarity of minor themes	32	Is there a description of diverse cases or discussion of minor themes?	9,10,11

Developed from: Tong A, Sainsbury P, Craig J. Consolidated criteria for reporting qualitative research (COREQ): a 32-item checklist for interviews and focus groups. *International Journal for Quality in Health Care*. 2007. Volume 19, Number 6: pp. 349 – 357

Once you have completed this checklist, please save a copy and upload it as part of your submission. DO NOT include this checklist as part of the main manuscript document. It must be uploaded as a separate file.

For peer review only

BMJ Open

Patient perspectives on the HIV continuum of care in London: a qualitative study of people diagnosed between 1986 and 2014

Journal:	*BMJ Open*
Manuscript ID	bmjopen-2017-020208.R3
Article Type:	Research
Date Submitted by the Author:	01-Mar-2018
Complete List of Authors:	Bruton, Jane; Imperial College Lodon, Department of Infectious Disease Epidemiology<br>Rai, Tanvi; Imperial College Lodon, Department of Infectious Disease Epidemiology<br>Day, Sophie; Imperial College Lodon, Department of Infectious Disease Epidemiology; Goldsmiths University of London, Anthropology<br>Ward, Helen; Imperial College London, Department of Infectious Disease Epidemiology
**Primary Subject Heading**:	HIV/AIDS
Secondary Subject Heading:	Qualitative research, Health services research, Patient-centred medicine
Keywords:	HIV & AIDS < INFECTIOUS DISEASES, QUALITATIVE RESEARCH, PATIENT EXPERIENCE, HIV CARE CONTINUUM

only

SCHOLARONE™
Manuscripts

Patient perspectives on the HIV continuum of care in London: a qualitative study of people diagnosed between 1986 and 2014

Jane Bruton RN, BA, MA Medical Anthropology; Tanvi Rai PhD Public Health, MPH, BA, BSc; Sophie Day, PhD, BA, MA Anthropology; BSc Helen Ward PhD, FRCP, FFPH, MBChB, MSc Epidemiology.

Corresponding author:
Pamela Jane Bruton,
Department of Infectious Disease Epidemiology,
School of Public Health,
Imperial College,
St Mary's Campus,
Norfolk Place,
London.
W2 1PG
jbruton@ic.ac.uk
0207 5943464

Co-authors:
Rai, Tanvi: Imperial College London, Department of Infectious Disease Epidemiology.
Day, Sophie: Imperial College London, Department of Infectious Disease Epidemiology and Goldsmiths College
Ward, Helen: Imperial College London, Department of Infectious Disease Epidemiology.

Word count: Main text 4051

For peer review only

For peer review only - http://bmjopen.bmj.com/site/about/guidelines.xhtml

Abstract

Objectives: To describe the experiences of the HIV treatment cascade of diagnosis, engagement with care and initiation of treatment, from the perspective of patients; we explored whether this differed according to the year of their diagnosis, for example whether they had experienced HIV care in the pre-treatment era.

Design: Qualitative interview study with framework analysis

Setting: Two large HIV adult outpatient clinics in central London.

Participants: 52 HIV positive individuals, 41 men, 11 women, purposively sampled to include people who had been diagnosed at different stages in the history of the epidemic classified as four 'generations': pre-1996 (pre-ART), 1997 to 2005 (complex ARTs), 2006 to 2012 (simpler ARTs), 2013 onwards (recent diagnoses).

Results: Some important differences were identified; for earlier generations, the visible illness and deaths from AIDS made it harder to engage with care following diagnosis. Subsequent decisions about starting treatment were deeply influenced by the fear of severe side-effects from early antiretroviral therapy (ART). However, despite improvements in ART and life expectancy over the epidemic we found a striking similarity across participants' accounts of the key stages of the care continuum, regardless of when they were diagnosed. Diagnosis was a major traumatic life event for almost everyone, fear of testing positive or having low self-perceived risk affected the timing of testing and diagnosis, engaging with care was facilitated by a flexible approach from services/clinicians and initiating treatment was a major life decision.

Conclusion: We found patients' experiences are influenced by when they were diagnosed, with earliest cohorts facing substantial challenges. However, being diagnosed with HIV and starting treatment continue to be significant life-altering events even in the era of effective, simple treatments. Despite the advances of biomedical treatment services should continue to recognise the needs of patients for whom the diagnosis and treatment remain significant challenges.

For peer review only

For peer review only - http://bmjopen.bmj.com/site/about/guidelines.xhtml

Strengths

- The large number of participants, at two different clinics and the inclusion of a range of people with HIV, broadly similar to the clinic cohorts, across four HIV 'generations'.
- The interview format allowed us to explore factors important to participants rather than asking about pre-defined concerns.

Limitations

- An imbalance between generations, with larger numbers of women in the earliest two generations and only one woman in the generation recently diagnosed. This means that some of our conclusions about generations may also reflect gendered differences.
- Our recruitment methods meant we were not able to explore the experiences of those who remain outside care.
- Limitation of the single interview format means people were having to recall past experiences, some of which were three decades ago. This may have introduced recall bias with subsequent experience colouring earlier reports.

For peer review only

For peer review only - http://bmjopen.bmj.com/site/about/guidelines.xhtml

INTRODUCTION

The Human Immunodeficiency Virus (HIV) continuum of care provides stepwise estimates for the stages of engagement in care for people living with HIV (PLHIV).(1) The Joint United Nations Programme on HIV and AIDS (UNAIDS) goal to end the Acquired Immune Deficiency Syndrome (AIDS) epidemic by 2020 stipulates a target of 90% of all people with HIV be diagnosed, of whom 90% are on antiretroviral therapy (ART), of whom 90% (73% of PLHIV) are virally suppressed.(2) The United Kingdom (UK) has one of the best outcomes for HIV treatment and care in the world with an estimated 78% of PLHIV having an undetectable viral load.(3, 4) However the statistics do not and cannot tell us about the patient experience of passing through these stages of care whether it be good or bad. The continuum essentially measures the success of programmes from a provider rather than a patient perspective.(5)

Flowers argues that there is a tension between the certainty and confidence of a linear HIV pathway, associated with ideas of clinical efficacy, and patient experiences of diagnosis and prognosis, which can be full of uncertainty.(4) With the evolution of modern ARTs we have witnessed the transformation of HIV from an acute life-threatening infection to a treatable chronic condition and a concurrent evolving of the care continuum. However, it is unclear whether this change is reflected in patients' own experiences of passing through each of the stages of care. For example, has the moment of diagnosis become any less traumatic, and have decisions about starting treatment become simpler for patients? Analysis of patient narratives, historically and currently, may help to highlight significant factors for patients in the care continuum. This is particularly important in a climate of National Health Service (NHS) restructuring in the UK, which has led to reductions in non-clinical services and streamlining of care. To this end we explored the patient perspectives on the care continuum. And we hypothesized that patients' experiences of, and engagement with, care would differ according to what point in the epidemic they were diagnosed.

METHODS

We undertook a qualitative study of people attending two public HIV clinics in London that have provided care since the start of the epidemic; they were also chosen for their large size and diversity, in terms of demographics. They are both specialist HIV clinics linked to sexual health (genitourinary medicine) services. Care is provided by physician-led multidisciplinary teams where patients have a named consultant. HIV care in the UK is free and open access, allowing patients to register at their clinic of choice. We used a purposive sampling method to recruit patients with a range of experiences. To reflect the evolution of ART, we identified four 'generations' according to time of diagnosis: pre-1996 (pre-ART), 1997 to 2005 (complex ARTs), 2006 to 2012 (simpler ARTs), 2013

For peer review only - http://bmjopen.bmj.com/site/about/guidelines.xhtml

onwards (recent diagnoses). Within each generation, we aimed to include people with a range of characteristics, including, gender, exposure, age and ethnicity. Participants were recruited opportunistically by researchers attending clinical services, and through fliers and digital advertising in clinical areas. Recruitment was periodically checked against the recruitment matrix and under-represented groups/strata targeted.

Patients were provided with information and gave written consent. Interviews took place in private rooms in or near the clinics or at the patient's home; they were recorded and transcribed, and lasted between 60 and 90 minutes. The interviews were semi-structured and carried out by one male (CH) and three female researchers (JB, TR, JR), three of whom had clinical backgrounds. The interviews were based on a topic guide (see supplementary file) informed by a focus group of PLHIV who assisted in designing the research. We invited participants to recall their initial diagnosis and describe key points in their HIV journey including testing, disclosure, support, engaging with care, starting treatment, medication adherence, work and social life. Field notes were written after the interviews.

Transcripts were uploaded to NVivo, a qualitative data analysis software package. Using Framework analysis, we developed key themes through a systematic process which involved reading, rereading, coding and summarising the transcripts; and subsequent in-depth analysis of the dataset.(6)Final themes were discussed in the research group (HW, JB, SD, TR) and further analysed in relation to the existing literature.

Ethical approval was obtained from the National Research Ethics Service (NRES) (reference number 14/WM/0147) in May 2014, and research governance approval obtained from the local sites.

RESULTS

Fifty-two patients were recruited, 25 at one clinic and 27 at the other. The sample included 41 men and 11 women; 37 men acquired HIV through sex with other men (MSM), the rest through heterosexual contact (n=14) or injection drug use (n=1). There were 11 in generation 1, 14 generation 2, 17 generation 3 and 10 generation 4. The characteristics of the study participants alongside those of the clinic population for 2014 are shown in Table 1.

Table 1. Study sample characteristics compared with the clinic cohorts

	Clinic A				Clinic B			
	Study sample		2014 Cohort		Study sample		2014 Cohort	
**Gender**								
Male	18	72.0%	2459	77.5%	23	85.2%	7743	90.3%
Female	7	28.0%	715	22.5%	4	14.8%	830	9.7%

For peer review only - http://bmjopen.bmj.com/site/about/guidelines.xhtml

**Age**								
18-24	0	0.0%	124	3.9%	0	0.0%	186	2.2%
25-34	4	16.0%	465	14.7%	3	11.1%	1729	20.2%
35-49	16	64.0%	1502	47.3%	14	51.9%	4164	48.6%
50+	5	20.0%	1083	34.1%	10	37.0%	2494	29.1%
**Ethnicity**								
White	14	56.0%	1541	48.6%	22	81.5%	6401	74.7%
Black African	5	20.0%	720	22.7%	5	18.5%	778	9.1%
Black Caribbean	0	0.0%	114	3.6%	0	0.0%	201	2.3%
Other/Mixed	6	24.0%	746	23.5%	0	0.0%	1132	13.2%
Not reported	0	0.0%	53	1.7%	0	0.0%	61	0.7%
**Exposure route**								
Sex between men	15	60.0%	1971	62.1%	22	81.5%	6776	79.0%
Heterosexual contact	9	36.0%	1037	32.7%	5	18.5%	1209	14.1%
Injecting drug use	1	4.0%	52	1.6%	0	0.0%	109	1.3%
Other	0	0.0%	112	3.5%	0	0.0%	46	0.5%
Undetermined	0	0.0%	2	0.1%	0	0.0%	433	5.1%
**Year of diagnosis**								
Pre-1997	6	24.0%	638	20.1%	5	18.5%	1399	16.3%
1997-2005	6	24.0%	1234	38.9%	8	29.6%	2580	30.1%
2006-2012	7	28.0%	986	31.1%	10	37.0%	3199	37.3%
2013 onwards	6	24.0%	316	10.0%	4	14.8%	1395	16.3%
Total			3174				8573	

The generation samples differed somewhat by gender and acquisition: the women were concentrated in generations 1 and 2 (n=6 and 4, respectively), and MSM in generations 3 and 4 (n=16 and 8, respectively).

We have used pseudonyms for each of the following quotes from participants.

"Becoming positive" – the impact of HIV diagnosis

The experience of receiving an HIV diagnosis was similar across the generations. First reactions were generally of shock and fear of death, irrespective of generation. Alan, diagnosed in 1991, knew nothing about HIV and had not tested before. He remembers vividly the time he received his result:

For peer review only - http://bmjopen.bmj.com/site/about/guidelines.xhtml

"I could hear myself saying 'I'm going to die'. Not verbally but in my mind, 'I'm going to die, I'm going to die'" (gen1, MSM)

Sylvia, diagnosed 2001, was "totally devastated":

"… I didn't see myself going back and doing my Master's degree for what reason am I going back to do that if I have maybe five years to live" (gen 2, woman)

Roger, diagnosed more than 20 years later, reported several previous tests and considered himself well-informed. However, his principal concern on receiving a positive diagnosis was also about life expectancy:

"But even I was not certain. Certainty is the wrong word. I was under the illusion that my expiry date was stamped on me now" (gen4, MSM)

Fear of a positive result was a key factor in delayed diagnosis for several MSM in all generations, who reported concerns about the impact of HIV on their lives. They were aware of their risk, and described feeling relieved at diagnosis as HIV had been *"hanging over them"* for years; the diagnosis confirmed their suspicions. William, who had never tested before, presented with symptoms:

"[I had been] burying my head in the sand. I guess I knew I had it but didn't, at the same time, want it confirmed" (gen2, MSM)

Brian (Gen4, MSM), recently diagnosed, had "*spent on and off probably eight years thinking about it*" He felt he had "*done all the thinking before*" … so although disappointed, he was also relieved.

Most other participants, particularly heterosexual men and women, were not expecting a positive result and had not requested an HIV test. They were diagnosed either following ongoing symptoms of ill health or having presented for a general sexual health check-up.

None of the women had been diagnosed during pregnancy; most were diagnosed before ante-natal screening became routine in the UK (1999). For example, Olivia, diagnosed in 1998, had not been tested in pregnancy. Her six-month old baby became sick, and both baby and husband were then diagnosed with HIV but she did not believe she had HIV and delayed testing for several weeks

"Me I don't have HIV because I never went with other men" (gen2, woman)

The response and level of support offered by clinicians at this critical time were important to participants' immediate wellbeing and influenced what happened next, including continuing engagement in care. Martha remarked:

"I remember how lovely [name of clinician] was and I've always said I could never wish for a better person to ever tell me or try to guide me, or to reassure me more than what she did because she was perfect" (Gen4, woman)

For peer review only - http://bmjopen.bmj.com/site/about/guidelines.xhtml

While most experiences were positive, there were some exceptions. Paul (gen3, MSM) had regularly tested negative but continued to take risks. Testing positive in 2010 was totally unexpected, leaving him "*numb with shock*", and he did not feel that he was supported appropriately. The clinician who gave him the diagnosis seemed "[to be on] *autopilot because he had seen people like me before*" and was "*working to his own agenda*". Despite Paul's obvious distress, the clinician asked him to ring potential contacts during the consultation. Further, when the clinician said: "*Oh this can be managed, don't worry*", Paul interpreted this to mean 'managed to his death'. After two weeks of acute anxiety, Paul contacted a friend who was able to reassure him about treatment and the care pathway. Similar experiences led other participants to feel vulnerable, isolated and slow to accept their diagnosis.

"Becoming an HIV patient": Developing a relationship with clinic and clinician

Once diagnosed, participants described a sense of reassurance about being in the "best hands*",* managed by experts in HIV medicine. The majority across the generations described strong relationships with their clinicians and valued seeing the same person each visit. It felt "*like a partnership*" with "*someone you can tell anything*", who knew them and their entire history, ensuring that care went beyond just the clinical management of HIV: "*We seriously talk about how I am not just what my CD4 count is*".

However, some had not developed a trusting relationship. Marty (gen3, MSM), for example, diagnosed HIV in 2012, was not eligible for treatment under guidelines at that point. He described anxiety about this lack of treatment, feeling that it adversely affected pre-existing mental health problems which were not addressed by his clinicians. He attended two different clinics and was on the verge of dropping out of care when, as he recounted: *"I basically rescued myself*". His friend recommended a clinician: "*She got me just like that thank god, thank god…I finally found and she was willing to fight my case*".

Another participant, Peter, diagnosed 2009, reported changing HIV clinics within three months of diagnosis. He recalled a series of mistakes, miscommunication and a "dehumanising" clinic environment. Losing trust in clinicians and the service, he finally gained confidence from attending a support group and moved his care:

> "*I remember that I said, it was like falling off a building… I'm slowly falling backwards as the virus increases. It felt like they were holding a blanket at the bottom to catch me but it felt like they were holding it in the wrong place. I was being asked to trust"* (gen3, MSM)

For peer review only - http://bmjopen.bmj.com/site/about/guidelines.xhtml

All participants valued continuity of care although two recently diagnosed felt that it was not always necessary to see the same clinician. But, continuity was affected by what some described as the very busy clinics limiting the time for consultations and impeding communication.

All participants were in care at the time of the interview but some described having stayed away in the past. Two of six women diagnosed before 1996 had dropped out of care for several years. Given the lack of effective treatment, they had found clinic visits depressing and preferred to keep away until they became sick. Marie (gen1, woman) explained, *"I didn't want a life where I just would go to tests and I am scared and they had nothing to offer".* Alison (gen1, woman) described the *"terrible situation"* at the clinic, where she saw young gay men, couples, where one would be fit and the other *"in a wheelchair, a skeleton".* She felt sorry for the doctors - *"there were all these poor young doctors with nothing to offer and seeing these very ill people.*"

Seven of all those diagnosed since ARTs became available described occasional or multiple lapses in attendance; these were generally explained by issues external to the clinic such as recreational drug use, household disruption, mental health problems or competing co-morbidities. Re-engagement with care was easier when their clinician actively reached out; for example, some consultants had telephoned patients when they missed appointments and one woman (gen1) described interventions of this kind as being "*like my family*". Even though not all patients were contacted when they did not attend services, and one expressed surprise that no-one had tried, all found their way back into care.

"Becoming medicalised": Starting treatment

Almost all (48) of the participants were on treatment; one had stopped medication because of drug interactions and three chose to remain off treatment. Despite the simplification of regimens, participants in all four generations found the decision to start treatment a significant life event.

For earlier generations, the decision had been complex because of toxicity related to ARTs. Some refused treatment contrary to medical advice. Alison, described earlier, recalled, "*In the waiting rooms people said, 'don't take it, it will kill you', so I refused that (AZT*)". Others felt well without treatment like Marie who was diagnosed in 1986 but only started ARTs 25 years after diagnosis when her CD4 count crashed. She felt she had no choice, "I fought it all this time on my own, and then finally I had to give in and take a pill. That was kind of depressing".

For peer review only - http://bmjopen.bmj.com/site/about/guidelines.xhtml

Those diagnosed more recently found it easier to decide to begin treatment, but it was still a significant moment. Tim, diagnosed in 2012, was aware of the latest research and asked to start treatment immediately, even for him, the 'treatment appointment' was a sobering experience:

> *"[It was] the only time there was a tear. I just thought, God this is a new chapter now. This is a new chapter in my life. I am going to have to take this pill for the rest of my life".* (, gen 3 MSM)

Brian, diagnosed in 2013, sums up some of the issues that participants said they had considered when deciding to start medication:

> *"Well the impact it would have on my life, the damage it would do to my body. Would I cope with the medication? Would I be able to continue working? Because so many people have side effects initially and it takes them a long time to get over. I had a lot of responsibility at work and I couldn't actually manage responsibility well enough once on medication. Would I be able to take the medication on time? Would life's pressures allow me to do what I needed to do? And so on"* (gen 4, MSM)

The three participants not on treatment described feeling healthy and wished to remain drug free for as long as possible.

For many participants, HIV occupied only a small part of their lives but daily medications proved to be a constant reminder of their status even if only for a moment each day. Martin, diagnosed in 2014, described his relationship with his medication:

> "*It's strange sometimes because you look at this pill and you think between you and this little pill lies – it's keeping you alive. And I have never had a pill to take like that before. So, it's very strange. It's my friend and foe at the same time"* (gen 4, MSM)

For peer review only

DISCUSSION

We have found that patients' experiences of, and engagement with, care are influenced by the point at which they were diagnosed, with the earliest cohorts facing substantial challenges. However, we have also found that being diagnosed with HIV and starting treatment continue to be significant life-altering events even in the era of effective and simple treatments. This study brings new insights which are important when considering how future services should be provided.

The revolution in HIV treatment over three decades means it can now be described as a chronic, manageable condition.(7, 8) In our study, all our patients were virally supressed having passed

For peer review only - http://bmjopen.bmj.com/site/about/guidelines.xhtml

through all the stages of the continuum and arrived on the other side. However, patients' recall of their experience of navigating this journey revealed a range of quite complex issues faced by them at different points, reminding us that many challenges remain in the successful provision of HIV care. We hypothesized that the revolution in treatment would have an impact on the experience of the different diagnostic generations moving through the care continuum, with those diagnosed more recently having a smoother journey. We identified some important differences; the visible illness and deaths from AIDS made it harder for earlier generations to engage with or remain in care and some dropped out, returning only when ill. Decisions about treatment were particularly difficult for the early generations, for whom the association of treatment with severe side effects remained strong until more recently and who expressed pride in, and determination to, remaining well without treatment.

However, our primary finding was a striking similarity across participants' accounts of key stages of the HIV care continuum: diagnosis was a major, traumatic life event for almost everyone, and anticipation of an HIV-positive status affected the timing of testing and diagnosis. Engagement with care was facilitated by a responsive, flexible approach on the part of services and clinicians, starting with the way the positive diagnosis was handled. Finally, initiating treatment was a major life decision even when recommended by protocol and considered straightforward by clinicians.

Despite the drive to normalise HIV testing through simplified sampling, reduced pre-test discussion and expanded test settings,(9, 10) (11) receiving an HIV diagnosis remained a significant shock for most participants irrespective of generation, sexuality or gender, as suggested elsewhere.(12-15) Moreover, we found that none of the heterosexual participants were expecting a positive result, a finding which also applied to some of the MSM participants. Resonating with other studies.(13, 16, 17) , we found that fear of imminent death and experience of profound distress did not change, despite the availability of effective and less toxic treatments. This fear, often coupled with a fear of social exclusion and rejection, led some MSM participants who suspected they were positive to delay testing.(18) Bury (1982) usefully describes this experience of illness and especially chronic illness as "biographical disruption". When everyday life and its meanings are turned upside down, relationships and social networks are disrupted and plans for the future have to be re-examined. (19) Participants described this type of disruption at diagnosis, whether HIV was considered an acute infection or a chronic condition.

A clinician's approach to a patient with a positive result is considered critical to patients' experiences and may be more important than other aspects of the testing process.(12, 15, 17, 20, 21) We found that negative experiences at this critical point affected immediate well-being and further contact

For peer review only - http://bmjopen.bmj.com/site/about/guidelines.xhtml

with services. The impact of those initial encounters, both good and bad, left participants with lasting impressions throughout their journey, thus demonstrating the importance of establishing trust between clinician and patient as a firm foundation for good retention in care.(22-24) Women, a minority in the clinics and, in our sample mainly from the earlier generations, faced particular challenges in engaging in care. This made the establishment of a trusting relationship with their clinician all the more important to managing their quality of life with HIV. Moreover, the importance of that relationship for participants was underlined by their willingness to change their treatment centre until they found what they perceived as a good clinician/patient relationship. The UK policy of open access to any clinic through self-referral may be another explanation for high levels of retention in care.

The prospect of treatment for life, to sustain life, was a major life event. Currently the moves towards starting treatment at diagnosis, the test-and-treat model, is based on the confidence of biomedicine in HIV management, but may be at odds with patient concerns.(25) British HIV Association (BHIVA) interim guidelines 2016 recommend starting treatment on all diagnosed with HIV regardless of CD4 count and continue to recognise that social, psychological, cultural, and economic factors can adversely affect adherence and treatment outcomes.(26) Starting medication on the same day as, or soon after, diagnosis when individuals may be distressed by the positive result could preclude a meaningful discussion of the patient's 'readiness to start'. Persson's 2016 study of patients not on ARTs found similar barriers and concerns: for example, logistics of starting life-long medication, fear of long term side effects and desire to stay drug-free whilst healthy.(27)

Considering ARTs was another point at which some participants anticipated 'biographical disruption' which deterred them from starting treatment.

The study's strengths are in the large number of participants at two different clinics, the inclusion of a range of people with HIV broadly similar to the cohorts seen at these clinics, and diagnosed across the four generations. The semi-structured interview format allowed us to explore factors important to participants rather than asking about pre-defined concerns. However, our study sample was imbalanced between generations, with larger numbers of women in the earliest two generation and only one of those more recently diagnosed. This means that some of our conclusions about generation may also reflect gendered differences. Our recruitment methods that relied on recruitment from the two clinics meant we were not able to explore the experiences of those who remain outside care. The study is also limited by the single interview format which meant people were having to recall past experiences, some of which were three decades ago. This may have introduced recall bias with subsequent experience colouring earlier reports; moreover, earlier

For peer review only - http://bmjopen.bmj.com/site/about/guidelines.xhtml

generations had more time to have disengaged and re-engaged with care. The focus of the study on a particular model of care in London limits the generalisability of our findings to other settings.

The evolution of simpler treatments has been accompanied by a reconfiguration of the care pathway. In London, HIV clinics are increasingly narrowly focused on HIV and HIV-specific medications and clinicians are not authorised to provide more holistic medical care.(28) In practice, this has led to less frequent clinic visits, a shift to virtual “e-clinics”, and greater links with, and reliance on, general practitioners. The impact of this on the care continuum is unclear. Continuity of clinician, the atmosphere in the clinic and good communication are recognised to be key issues for patients. (29, 30)

There is increasing recognition that viral suppression is not the final goal for people who are now living longer with HIV.(31-33) Lazarus et al (2016) have called for a ‘fourth 90’ “providing an explicit target for health- related quality of life”(31). In December2017 these concerns were embodied in policy recommendations from the European Parliament calling for an integrated and patient-centred approach to long term HIV care ensuring that services are meeting this challenge. (34) Our study illustrates that the patient journey is complex and personalised care should not be lost with streamlining pathways. All our participants were virally suppressed but that can hide the reality of their reliance on the caregivers to help maintain that stability.

A personal, holistic approach has been a hallmark of HIV care since the beginning of the epidemic. It is important that major advances in biomedical treatment do not undermine the care continuum through a loss of care that meets the complex needs of patients, for whom HIV diagnosis and treatment remain significant challenges requiring supportive and flexible care.

For peer review only

For peer review only - http://bmjopen.bmj.com/site/about/guidelines.xhtml

Acknowledgements: Thanks to participants for sharing their time and stories, to Jane Rowlands and Chris Higgs for conducting some of the interviews and the staff of both clinics for facilitating the study.

Contributership statement: All four authors, J. Bruton, T. Rai, S. Day and H. Ward, were involved in the design of the study. J. Bruton and T. Rai completed the initial analysis and interpretation of the data and subsequent discussion and analysis was undertaken by all 4 authors through team discussion. J. Bruton completed the first draft of the manuscript. All four authors participated in critiquing and revising the draft manuscript and all approved the final draft.
All four authors accept accountability for the submitted paper.

Collaborators: J. Rowlands and C.Higgs undertook some of the interviews at one of the two study sites.

Funders: Funded by grants from Imperial NIHR Biomedical Research Centre, P35771, the Imperial College Healthcare Charity, P44223, and supported by the St Stephens AIDS Trust. No pharmaceutical grants were received in the development of this study.

Competing Interests: There are no competing interests

Data sharing statement: The interview transcripts are anonymised and stored securely at Imperial College London on a password protected database on a password protected computer. The participants were assured of the security of their data and they would remain confidential.

References

1. Gardner EM, McLees MP, Steiner JF, del Rio C, Burman WJ. The Spectrum of Engagement in HIV Care and its Relevance to Test-and-Treat Strategies for Prevention of HIV Infection. Clinical Infectious Diseases. 2011;52(6):793-800.
2. UNAIDS. UNAIDS. 90-90-90 - an ambitious treatment target to help end the AIDS epidemic (accessed June 21, 2017) http://www.unaids.org/en/resources/documents/2014/90-90-90. 2014.
3. Kirwan PD CC, Brown AE., Gill ON.,Delpech VC. and contributors. HIV in the UK: 2016 Report. London: Public Health England; 2016.
4. Flowers P, Davis MM, Larkin M, Church S, Marriott C. Understanding the impact of HIV diagnosis amongst gay men in Scotland: an interpretative phenomenological analysis. Psychology & health. 2011;26(10):1378-91.
5. Christopoulos KA, Massey AD, Lopez AM, Geng EH, Johnson MO, Pilcher CD, et al. "Taking a half day at a time:" patient perspectives and the HIV engagement in care continuum. AIDS patient care and STDs. 2013;27(4):223-30.
6. Gale NK, Heath G, Cameron E, Rashid S, Redwood S. Using the framework method for the analysis of qualitative data in multi-disciplinary health research. BMC Medical Research Methodology. 2013;13(1):117.

For peer review only - http://bmjopen.bmj.com/site/about/guidelines.xhtml

7. Deeks SG, Lewin SR, Havlir DV. The End of AIDS: HIV Infection as a Chronic Disease. Lancet. 2013;382(9903):1525-33.
8. Yehia BR, Gebo KA, Hicks PB, Korthuis PT, Moore RD, Ridore M, et al. Structures of Care in the Clinics of the HIV Research Network. AIDS patient care and STDs. 2008;22(12):1007-13.
9. Ogaz D. FM, Connor N., Gill ON. and contributors. HIV testing in England:2016 report. London England: Public Health England; 2016.
10. Rayment M, Thornton A, Mandalia S, Elam G, Atkins M, Jones R, et al. HIV Testing in Non-Traditional Settings – The HINTS Study: A Multi-Centre Observational Study of Feasibility and Acceptability. PLOS ONE. 2012;7(6):e39530.
11. Wise J. Guidelines call for HIV testing to be "normalised". BMJ. 2008;337.
12. Roth NL, Nelson MS. HIV diagnosis rituals and identity narratives. AIDS Care. 1997;9(2):161-80.
13. Stevens PE, Hildebrandt E. Life changing words: women's responses to being diagnosed with HIV infection. ANS Advances in nursing science. 2006;29(3):207-21.
14. Stevens PE, Tighe Doerr B. Trauma of discovery: women's narratives of being informed they are HIV-infected. AIDS Care. 1997;9(5):523-38.
15. Anderson M, Elam G, Gerver S, Solarin I, Fenton K, Easterbrook P. "It took a piece of me": initial responses to a positive HIV diagnosis by Caribbean people in the UK. AIDS Care. 2010;22(12):1493-8.
16. Meursing K, Sibindi F. HIV counselling — a luxury or necessity? Health Policy and Planning. 2000;15(1):17-23.
17. Christopoulos KA, Massey AD, Lopez AM, Hare CB, Johnson MO, Pilcher CD, et al. Patient Perspectives on the Experience of Being Newly Diagnosed with HIV in the Emergency Department/Urgent Care Clinic of a Public Hospital. PLOS ONE. 2013;8(8):e74199.
18. Clifton S, Nardone A, Field N, Mercer CH, Tanton C, Macdowall W, et al. HIV testing, risk perception, and behaviour in the British population. AIDS (London, England). 2016;30(6):943-52.
19. Bury M. Chronic illness as biographical disruption. Sociology of health & illness. 1982;4(2):167-82.
20. Worthington C, Myers T. Desired elements of HIV testing services: test recipient perspectives. AIDS patient care and STDs. 2002;16(11):537-48.
21. Hult JR, Maurer SA, Moskowitz JT. "I'm sorry, you're positive": a qualitative study of individual experiences of testing positive for HIV. AIDS Care. 2009;21(2):185-8.
22. Kempf MC, McLeod J, Boehme AK, Walcott MW, Wright L, Seal P, et al. A qualitative study of the barriers and facilitators to retention-in-care among HIV-positive women in the rural southeastern United States: implications for targeted interventions. AIDS patient care and STDs. 2010;24(8):515-20.
23. Yehia BR, Stewart L, Momplaisir F, Mody A, Holtzman CW, Jacobs LM, et al. Barriers and facilitators to patient retention in HIV care. BMC Infectious Diseases. 2015;15(1):246.
24. Engelhard EA, Smit C, Van Sighem A, Reiss P, Nieuwkerk PT, Kroon FP, et al. Impact of HIV care facility characteristics on the cascade of care in HIV-infected patients in the Netherlands. AIDS (London, England). 2016;30(2):301-10.
25. Newman CE. Appreciating doubts about HIV medicine2015.
26. BHIVA. British HIV Association guidelines for the treatmnet of HIV-1- positive adults with antiretroviral therapy. 2015 (2016 interim update) 2016.

27. Persson A, Newman CE, Mao L, de Wit J. On the Margins of Pharmaceutical Citizenship: Not Taking HIV Medication in the "Treatment Revolution" Era. Medical anthropology quarterly. 2016;30(3):359-77.
28. Miller R. GJ. Commissioning London's HIV Services. Civitas (Institute for the Study of Civil Society); 2011.
29. Weatherburn P. KP, Reid D., Hammond G., Jessup K. My care I care: A study of what people with HIV value about NHS HIV Services across London. Sigma Research; 2013.

For peer review only - http://bmjopen.bmj.com/site/about/guidelines.xhtml

30. Haggerty JL, Reid RJ, Freeman GK, Starfield BH, Adair CE, McKendry R. Continuity of care: a multidisciplinary review. BMJ: British Medical Journal. 2003;327(7425):1219.
31. Lazarus JV, Safreed-Harmon K, Barton SE, Costagliola D, Dedes N, del Amo Valero J, et al. Beyond viral suppression of HIV – the new quality of life frontier. BMC Medicine. 2016;14(1):94.
32. Highlights from the BHIVA Satellite Symposium, IAS Conference, Paris, France, July 2017: 'Tougher times: adapting to increasing demand with declining resources'. Journal of Virus Eradication. 2017;3(4):250-2.
33. Martel K CJ, Auberbach J. Looking Beyond Viral Suppression: Findings from The Well Project's User Survey on Factors Influeincing the Health, Well-being, and Quality of Life of Women Living with HIV [IAS 2017 Poster]. 2017 [Available from: http://www.thewellproject.org/news-press/looking-beyond-viral-suppression-findings-well-project%E2%80%99s-2016-user-survey-factors.
34. HIV Outcomes, Beyond Viral Suppression. Recommendations Launched at the European Parliament. 2017 [cited 03/01/2018]. [cited 03/01/2018]. Available from: http://hivoutcomes.eu/wp-content/uploads/2017/11/HIV_Booklet_FINAL-DIGITAL-version.pdf.

For peer review only

For peer review only - http://bmjopen.bmj.com/site/about/guidelines.xhtml

Key statements

- A qualitative study understanding the patients' perspective of their experience of the HIV care continuum
- The diverse study cohort spans 4 'generations' diagnosed at different time points between 1986 and 2014 reflecting the evolution of ARTs
- There are striking similarities of experience and significance at key points on the continuum: diagnosis, engaging in care and starting treatment
- Despite advances of biomedical treatment clinical services should continue to recognise the needs of patients for whom HIV diagnosis and treatment remains a significant challenge

For peer review only

For peer review only - http://bmjopen.bmj.com/site/about/guidelines.xhtml

Imperial College London

Chelsea and Westminster Hospital NHS
NHS Foundation Trust

Imperial College Healthcare NHS
NHS Trust

<u>HIV patient interview topic guide</u>

Overview description (recap and summary of information in the patient information sheet)

During this project we wish to find out people's own experiences of their life with HIV starting from the beginning, before you were diagnosed and going all the way up to the present. There are some specific areas we wish to cover, however you are free to discuss what you feel are important issues or areas that have been relevant in your HIV journey.

Questions:

[May or may not need to ask all the questions specifically, this is just a guide, some questions overlap]

1. Personal information – work/relationships/home situation

Can you tell me a bit about yourself….

a. What are your home circumstances?
b. Are you in a relationship/s?
c. Do you have dependents children? Parents? partner ?
d. Do you work? What do you do? Benefits?

2. Testing decisions

a. Can you tell me when you were diagnosed?
b. How did you come to be tested?
c. Where did you go for your test? Why?
d. Had you had HIV tests before, if so why?
e. What was your health like before your diagnosis?
f. Had/did you know anybody living with HIV at the time?

3. Experience of initial care

a. What happened when you were given your positive result?
b. Can you remember what information you were given at the time? Was this given in the way you wanted? Please can you tell me in detail.
c. What questions, if any, did you have for the clinical staff? Did you ask them?

For peer review only - http://bmjopen.bmj.com/site/about/guidelines.xhtml
For peer review only

d. Did you tell anyone / talk to anyone after you found out your results? Can you tell me a bit more about this – who were they, was it helpful to talk to them, etc.?
e. Did you access information about HIV? How? where? How has this helped?
f. What did you do after receiving your diagnosis?
g. Which HIV clinic did you go to? Why? Are you still at that clinic? Why?

4. Treatment decisions and research

a. Are you on treatment ?
b. How was the decision to start taken? Were you involved in that decision?
c. What preparation did you make?

d. (If not on treatment) What are your thoughts about going on treatment?

(If on treatment) What do you think / have you any thoughts about being on treatment?

e. (If on treatment) How do you find taking your medication?

f. Have you discussed your thoughts about treatment with anyone?
g. Do you know what recommendations there are for when people might start treatment?

h. Are you aware of the research into ARVs and treatment guidelines in the UK?
i. What do you know about them? What do you think of them?
j. How have they influenced your understanding of treatment?
k. Have they influenced your decision to start/change/stop treatment?

l. Do you take part in clinical trials? Why?

5. Clinical care

a. How often do you go to the clinic? Has that changed over the time you have been in the clinic? How?
b. Who do you see? And for what? Doctor? Consultant? Nurse? Peer support? Health Advisor? etc
c. What kind of care have you received at the clinic? Any good/bad experiences?

d. What do you think the role of the clinic is in your care?
e. What other clinical services do you access? Where? How is your care coordinated?
f. What do you think about the way in which HIV services are currently organised? Does it work for you?
g. Have they changed? In what way?
h. Are you aware of possible future changes in the services?

For peer review only - http://bmjopen.bmj.com/site/about/guidelines.xhtml
For peer review only

6. Primary care /Community and Social care

a. Where do you go for your primary care needs? Why? What is your experience of primary care?
b. How is your care coordinated between primary care and the clinic?
c. Do you access any social care or voluntary services? If so what? Why?
d. Do they meet your needs? If not why?
e. Have you seen any changes in what is available? If so what?

7. Living with HIV

a. Has having HIV affected your life? What are the most significant ways HIV has affected your life, and has that changed over time?
b. Do you know others living with HIV? What impact does that have on your life?
c. What kind of support have you had/needed since you were diagnosed positive? Has this changed over time?

8. Is there anything else you would like to say?

a. Given your experience of living with HIV, what would you say to others who have been recently diagnosed about what to expect?

For peer review only

For peer review only - http://bmjopen.bmj.com/site/about/guidelines.xhtml

COREQ (COnsolidated criteria for REporting Qualitative research) Checklist

A checklist of items that should be included in reports of qualitative research. You must report the page number in your manuscript where you consider each of the items listed in this checklist. If you have not included this information, either revise your manuscript accordingly before submitting or note N/A.

Topic	Item No.	Guide Questions/Description	Reported on Page No.
**Domain 1: Research team and reflexivity**			
Personal characteristics			
Interviewer/facilitator	1	Which author/s conducted the interview or focus group?	4
Credentials	2	What were the researcher's credentials? E.g. PhD, MD	1
Occupation	3	What was their occupation at the time of the study?	1
Gender	4	Was the researcher male or female?	1
Experience and training	5	What experience or training did the researcher have?	1
Relationship with participants			
Relationship established	6	Was a relationship established prior to study commencement?	4
Participant knowledge of the interviewer	7	What did the participants know about the researcher? e.g. personal goals, reasons for doing the research	4
Interviewer characteristics	8	What characteristics were reported about the inter viewer/facilitator? e.g. Bias, assumptions, reasons and interests in the research topic	1
**Domain 2: Study design**			
Theoretical framework			
Methodological orientation and Theory	9	What methodological orientation was stated to underpin the study? e.g. grounded theory, discourse analysis, ethnography, phenomenology, content analysis	5
Participant selection			
Sampling	10	How were participants selected? e.g. purposive, convenience, consecutive, snowball	4
Method of approach	11	How were participants approached? e.g. face-to-face, telephone, mail, email	4
Sample size	12	How many participants were in the study?	5
Non-participation	13	How many people refused to participate or dropped out? Reasons?	NA
Setting			
Setting of data collection	14	Where was the data collected? e.g. home, clinic, workplace	4
Presence of non-participants	15	Was anyone else present besides the participants and researchers?	4
Description of sample	16	What are the important characteristics of the sample? e.g. demographic data, date	5/6
Data collection			
Interview guide	17	Were questions, prompts, guides provided by the authors? Was it pilot tested?	5
Repeat interviews	18	Were repeat inter views carried out? If yes, how many?	NA
Audio/visual recording	19	Did the research use audio or visual recording to collect the data?	4
Field notes	20	Were field notes made during and/or after the inter view or focus group?	5
Duration	21	What was the duration of the inter views or focus group?	4
Data saturation	22	Was data saturation discussed?	N/A
Transcripts returned	23	Were transcripts returned to participants for comment and/or	NA

For peer review only

For peer review only - http://bmjopen.bmj.com/site/about/guidelines.xhtml

Topic	Item No.	Guide Questions/Description	Reported on Page No.
		correction?	
**Domain 3: analysis and findings**			
Data analysis			
Number of data coders	24	How many data coders coded the data?	5
Description of the coding tree	25	Did authors provide a description of the coding tree?	NA
Derivation of themes	26	Were themes identified in advance or derived from the data?	5
Software	27	What software, if applicable, was used to manage the data?	5
Participant checking	28	Did participants provide feedback on the findings?	NA
Reporting			
Quotations presented	29	Were participant quotations presented to illustrate the themes/findings? Was each quotation identified? e.g. participant number	6,7,8,9
Data and findings consistent	30	Was there consistency between the data presented and the findings?	9,10,11
Clarity of major themes	31	Were major themes clearly presented in the findings?	9,10,11
Clarity of minor themes	32	Is there a description of diverse cases or discussion of minor themes?	9,10,11

Developed from: Tong A, Sainsbury P, Craig J. Consolidated criteria for reporting qualitative research (COREQ): a 32-item checklist for interviews and focus groups. *International Journal for Quality in Health Care*. 2007. Volume 19, Number 6: pp. 349 – 357

Once you have completed this checklist, please save a copy and upload it as part of your submission. DO NOT include this checklist as part of the main manuscript document. It must be uploaded as a separate file.

For peer review only
